# Genetically Engineered Liposwitch-Based Nanomaterials

**DOI:** 10.1021/acs.biomac.4c01388

**Published:** 2024-11-04

**Authors:** Md Shahadat Hossain, Alex Wang, Salma Anika, Zhe Zhang, Davoud Mozhdehi

**Affiliations:** Department of Chemistry, Syracuse University, 111 College Place, Syracuse, New York 13244, United States

## Abstract

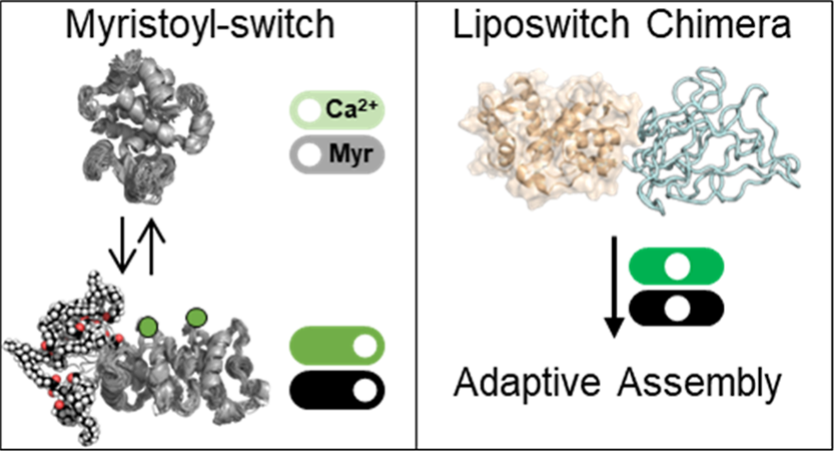

Fusion of intrinsically disordered and globular proteins is a powerful strategy to create functional nanomaterials. However, the immutable nature of genetic encoding restricts the dynamic adaptability of nanostructures postexpression. To address this, we envisioned using a myristoyl switch, a protein that combines allostery and post-translational modifications—two strategies that modify protein properties without altering their sequence—to regulate intrinsically disordered protein (IDP)-driven nanoassembly. A typical myristoyl switch, allosterically activated by a stimulus, reveals a sequestered lipid for membrane association. We hypothesize that this conditional exposure of lipids can regulate the assembly of fusion proteins, a concept we term “liposwitching”. We tested this by fusing recoverin, a calcium-dependent myristoyl switch, with elastin-like polypeptide, a thermoresponsive model IDP. Biophysical analyses confirmed recoverin’s myristoyl-switch functionality, while dynamic light scattering and cryo-transmission electron microscopy showed distinct calcium- and lipidation-dependent phase separation and assembly. This study highlights liposwitching as a viable strategy for controlling DP-driven nanoassembly, enabling applications in synthetic biology and cellular engineering.

## 1 Introduction

Gene fusion, the recombination of distinct genes to create multifunctional proteins, acts as a creative force in evolutionary biology by allowing organisms to merge functional characteristics of individual proteins and adapt to environmental changes.^1^ Advances in genetic engineering have rendered gene fusion a powerful strategy for merging the functional properties of proteins with the versatility of nanomaterials.^2−4^ Its major advantages lie in the genetic encoding that predetermines the nanoscale organization of proteins through sequence- and domain-specific interactions^5−12^ and the simpler, more cost-effective recombinant expression of fusion proteins compared to the conjugation of proteins to nanomaterials.^13−15^ These strengths have propelled the use of globular–globular fusions in various applications, including drug delivery,^16,17^ vaccine development,^18,19^ theranostics,^20^ tissue engineering,^21,22^ and enzyme engineering,^23−25^ particularly in scenarios where control of nanoscale structure is critical for function.^26−29^

Despite these strengths, the engineering of fusion proteins that can adaptively assemble in response to environmental stimuli continues to be a challenging yet crucial area of focus in the field.^30−37^ Intrinsically disordered proteins (IDPs) have emerged as potential building blocks for these stimuli-responsive nanomaterials.^38−41^ Unlike globular proteins, IDPs are conformationally flexible and lack a fixed structure due to their biased amino acid composition and low sequence complexity.^42^ This characteristic allows the assembly of globular-IDP fusions to be fine-tuned in response to environmental variables without altering their primary sequence.^43,44^ This fine-tuning is possible because IDPs can be programmed to interact through weak and dynamic noncovalent interactions that depend on physicochemical parameters and biological factors such as concentration, pH, temperature, ionic strength, and binding partners, among others.^45^ Despite impressive progress in developing responsive IDPs,^46^ a fundamental problem remains: the assembly of these fusions is fundamentally limited by their fixed genetic blueprint, significantly hindering the adaptability of these assemblies—after translation—and restricting the range of functions they can perform.^47^ New strategies to expand the properties of globular-IDP fusions would improve our ability to engineer, control, and program their nanoassembly toward new capabilities.^48^

**Figure 1:**
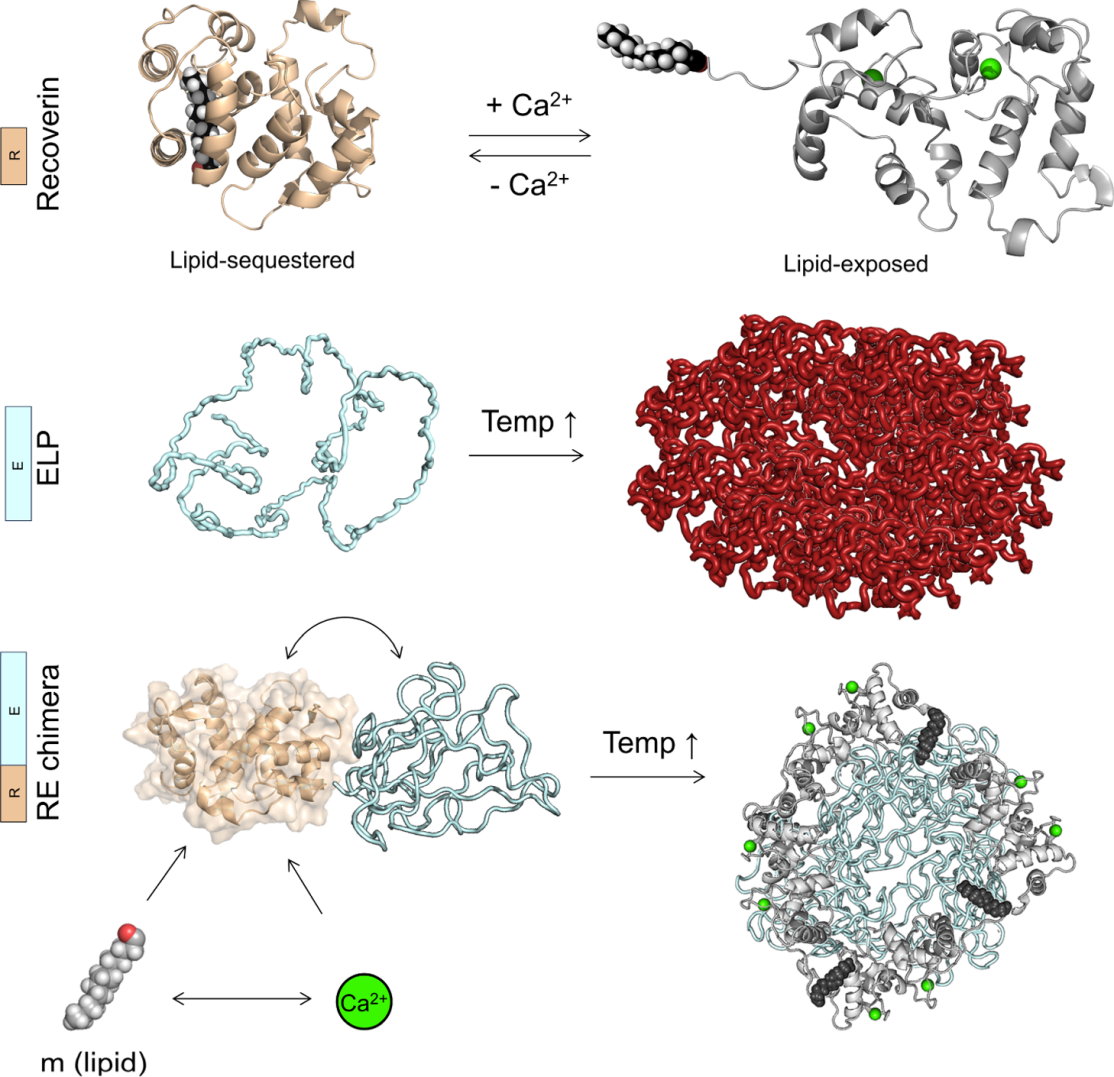
Schematic of our strategy for integrating post-translational modifications and allostery as mechanisms that drive external stimulus-responsive IDP-driven nanoassembly. Diagram of the constitutive domains: recoverin (R) as a myristoyl switch, ELP (E) as a thermoresponsive coil protein, and RE fusion. Recoverin transitions from a lipid-sequestered state to a lipid-exposed state upon Ca^2+^ binding (PDB ids 1IKU and 1JSA), and ELP undergoes temperature-induced aggregation.

Recognizing the potential of combining allostery with PTMs to (re)program the assembly of fusion proteins, this work focuses on proteins known as myristoyl switches, which embody both features. Myristoyl switches, such as recoverin, are allosteric proteins bearing a myristoyl (m) lipid PTM.^49−52^ In the absence of calcium, recoverin hides its lipid anchor in a hydrophobic pocket. Upon calcium binding, the lipid anchor is exposed, enabling recoverin to bind and localize to the membrane, thus triggering downstream effects (Figure 1).^50^ We hypothesize that harnessing the conditional exposure of lipid moieties allows for dynamic regulation of fusion protein assembly, a concept we call “liposwitching”. Our hypothesis builds on the understanding that calcium binding triggers allosteric conformational changes in recoverin, causing a 45-degree rotation of the N-terminal domain.^53^ When myristoylated, recoverin’s allosteric shift exposes the hydrophobic lipid, bringing it near the C-termini fusion and thereby influencing adjacent protein hydration, conformation, and the energy landscape.^54^ If this holds true, “liposwitching” would effectively combine and transduce allosteric conformational shifts and PTMs—enabling dynamic regulation of the fusion protein’s assembly and function—without modifying the protein sequence.

To test our hypothesis, we developed a fusion protein that combines recoverin with a model IDP. This design leverages recoverin’s liposwitching mechanism—unveiling hydrophobic motifs in response to Ca^2+^—to modulate the aggregation of the IDP in response to biologically relevant signals such as myristoylation and calcium binding. As a model IDP, we chose elastin-like polypeptides (ELPs).^55^ ELPs are artificial IDPs that consist of repeating units of the VPGXG pentapeptide (where X is a variable, nonproline amino acid) and exhibit reversible phase separation and assembly triggered by changes in temperature, pH, and other factors.^56^ Biophysical characterizations confirmed the integrity and functionality of recoverin’s myristoyl switch within the recoverin-ELP (RE) fusion protein. Dynamic light scattering (DLS) and cryo-transmission electron microscopy (cryo-TEM) demonstrated that, unlike traditional globular-IDP fusions responding to a single stimulus, RE fusions uniquely combine multiple responsive elements, with their temperature-triggered IDP-driven nanoassembly influenced by the interplay between myristoylation and calcium. Taken together, these results provide a foundational framework for IDP-based nanoassembly processes that can be dynamically regulated by cellular signals. This dynamic adaptability opens up new possibilities for integrating nanobiomaterials with cellular processes and chemistry, paving the way for advanced applications in synthetic biology and biotechnology.

## 2 Experimental Section

A detailed description of materials, cloning procedures, protein expression, purification, characterization, and fluorescent labeling is provided in the Supporting Information.

### 2.1 Sample Preparation

Lyophilized protein was dissolved in TBS at 4 °C to prepare the stock solution. Separate stock solutions of CaCl_2_ and EGTA were also prepared in TBS at a concentration of 100 mM. The protein stock solution’s concentration was confirmed using UV–vis spectroscopy at 280 nm using an extinction coefficient (ε) of 25,440 M^–1^ cm^–1^ (calculated using ProtParam). This concentrated stock was diluted to the required protein concentration for each characterization technique (details provided below) and supplemented with either CaCl_2_ (+Ca^2+^) or EGTA (−Ca^2+^) to a final concentration of 1 mM. All protein samples in this study were prepared using this method, except for those used in far-UV circular dichroism (CD) analysis. For far-UV CD, samples were prepared in Tris buffer rather than TBS to minimize chloride ion interference at wavelengths below 200 nm.

### 2.2 Circular Dichroism

Spectra were recorded on an Aviv model 420 CD spectrometer at 15 °C (*T* < *T*_E_) and processed using Aviv software v3.47. This temperature was selected to prevent the phase separation of RE constructs, which could result in the formation of larger assemblies that scatter light and interfere with data accuracy. For far-UV CD, protein solutions were prepared at concentrations of 2.5 μM (for RE and m-RE) and 5 μM (for R and m-R), and the CD signal was collected using a 1 mm quartz cell at 190–320 nm. The background spectrum, obtained using the buffer alone, was subtracted from each protein spectrum prior to converting the data into mean molar residue ellipticity (in deg cm^2^ dmol^–1^). Data deconvolution was performed by using DichroWeb with the CONTIN model. For near-UV CD measurements (250–320 nm), aimed at probing the environment of aromatic residues, the protein concentrations were increased to 40 μM, and the path length was adjusted to 5 mm to enhance the signal-to-noise ratio, thereby improving the detection of subtle spectral features.

### 2.3 Tryptophan Autofluorescence

Fluorescence spectra were recorded by using a Cary 100 eclipse spectrofluorometer (Agilent) at room temperature. Protein solutions were prepared at a concentration of 1 μM. The fluorescence emissions were recorded from 300 to 400 nm while exciting the samples at 290 nm.

### 2.4 Variable-Temperature Turbidimetry

The temperature-dependent phase behavior of proteins was investigated by using a Cary 300 UV–vis spectrophotometer (Agilent) equipped with a Peltier temperature controller. Turbidity was monitored by recording the absorbance at 350 nm across a temperature range of 15–55 °C, with a ramp rate of 1 °C/min. Fusion protein (RE) and ELP samples were tested at concentrations of 1, 5, 10, 25, 50, 75, and 100 μM (Figure S6). Due to its limited solubility, recoverin was analyzed at concentrations of 1, 5, 10, 25, and 50 μM. Transition temperatures were determined by analyzing the first derivative of the turbidity plots and identifying the inflection points of the turbidimetry profiles (Table S3).

### 2.5 Thermal Shift Assay

Thermal shift assays (differential scanning fluorometry) were conducted by using a QuantStudio 3 real-time PCR system (Thermo Fisher Scientific). Samples for each construct were prepared at a concentration of 1 mg/mL of protein in TBS containing 1 mM EGTA (−Ca^2+^) or 1 mM CaCl_2_ (+Ca^2+^) and 5× SYPRO Orange dye (Invitrogen). The solutions were centrifuged at 10,000 rpm for 1 min at 4 °C. After centrifugation, 25 μL of each sample was loaded into optical PCR tubes in triplicate. Control samples included proteins without dye and dye only in the buffer (no protein). The samples were equilibrated at 5 °C for 1 min and then heated to 98 °C at a rate of 1 °C/min. Fluorescence was monitored throughout the temperature ramp, and the fluorescence and d*F*/d*T* results were plotted as a function of temperature by using GraphPad Prism (version 9.5.1).

### 2.6 Dynamic Light Scattering

Variable-temperature dynamic light scattering (VT-DLS) experiments were performed by using a NanoLab 3D instrument (LSI Instrument) equipped with a 90° detector in a 3D cross-modulated geometry. Prior to DLS analysis, protein solutions (10 μM) were filtered through a 0.22 μm PES filter (Millipore Millex) and chilled at 4 °C. VT-DLS measurements were carried out over a temperature range of 16–54 °C, increasing in 2 °C increments with a 1 min equilibration period at each temperature. Each measurement involved three 20 s repetitions, with the instrument’s automatic attenuation feature adjusting laser intensity to achieve a scattering intensity of 250 kHz. The acquired correlation functions were analyzed using the cumulant algorithm in the LSLab software.

### 2.7 Confocal Microscopy

Protein structures were visualized using a Zeiss LSM 980 with an Airyscan 2 confocal microscope equipped with a 480 nm laser light source and a 20× objective. The microscope featured a heated stage and an environmental chamber to ensure consistent temperature control. Protein samples (30 μM, with 10% labeled) were placed in an Abidi 8-well glass microscope slide and incubated at each designated temperature for 10 min prior to imaging. The solution’s temperature was continuously monitored with a thermocouple (TCI Electronics) placed in a buffer-filled adjacent well. Image stacks from confocal microscopy were analyzed and merged using Fiji software, applying the *Z*-stack function and the maximum intensity projection algorithm for reconstruction.

### 2.8 Cryo-Transmission Electron Microscopy

Fresh protein solutions were prepared in TBS at 4 °C and incubated at 35 °C for 10 min. Postincubation, the samples were deposited onto freshly plasma-cleaned Quantifoil grids (Quantifoil Micro Tools GmbH, Germany) maintained in a Mk IV Vitrobot (Thermo Fisher Scientific) with 100% humidity. Vitrification was achieved by plunging the grids into liquid ethane with subsequent storage under liquid nitrogen. Imaging was conducted on a Tecnai BioTwin 120 kV transmission electron microscope equipped with a Gatan SC1000A CCD camera and operated at liquid nitrogen temperature. Low-dose imaging was performed by using Gatan 626 or 910 holders. Image processing was carried out in ImageJ, initially applying the despeckle plugin (median filter) for denoising. For calcium-containing samples requiring enhanced clarity, additional processing was done using a fast Fourier transform (FFT) band-pass filter (up to 10 pixels, down to 400, no stripe suppression, with autoscale and saturation).

## 3 Results and Discussion

### 3.1 Recoverin in RE Fusion Retains Its Structural and Functional Properties

The design of the fusion protein—the orientation and identity of domains—was informed by the stability and functional requirements of recoverin. Recoverin’s myristic acid must be located at the N-terminus to function as a myristoyl switch. Therefore, we positioned the thermoresponsive ELP domain at the C-terminus (Figure 1). We selected a hydrophobic ELP, composed of 80 repeats of GVGVP, to ensure that its transition temperature remains sufficiently below that of recoverin and that IDP-driven assembly occurs at temperatures that do not affect the structural integrity of recoverin. Herein, we refer to “recoverin” and “ELP” as Rc and Ec, when expressed individually. When expressed as a fusion, we refer to them as RE and use the terms “R-domain” and “E-domain” to discuss these proteins within the context of the RE chimera. We also produced myristoylated constructs of Rc and RE using an established procedure (see Experimental Section and Supporting Information).^57,58^ After purification, all constructs were characterized with SDS–PAGE (Figure S1), RP-HPLC, MALDI–TOF, and trypsin-digest LC–MS to confirm their identity and ensure purity greater than 95% (Figures S2 and S3 and Table S1).

**Figure 2:**
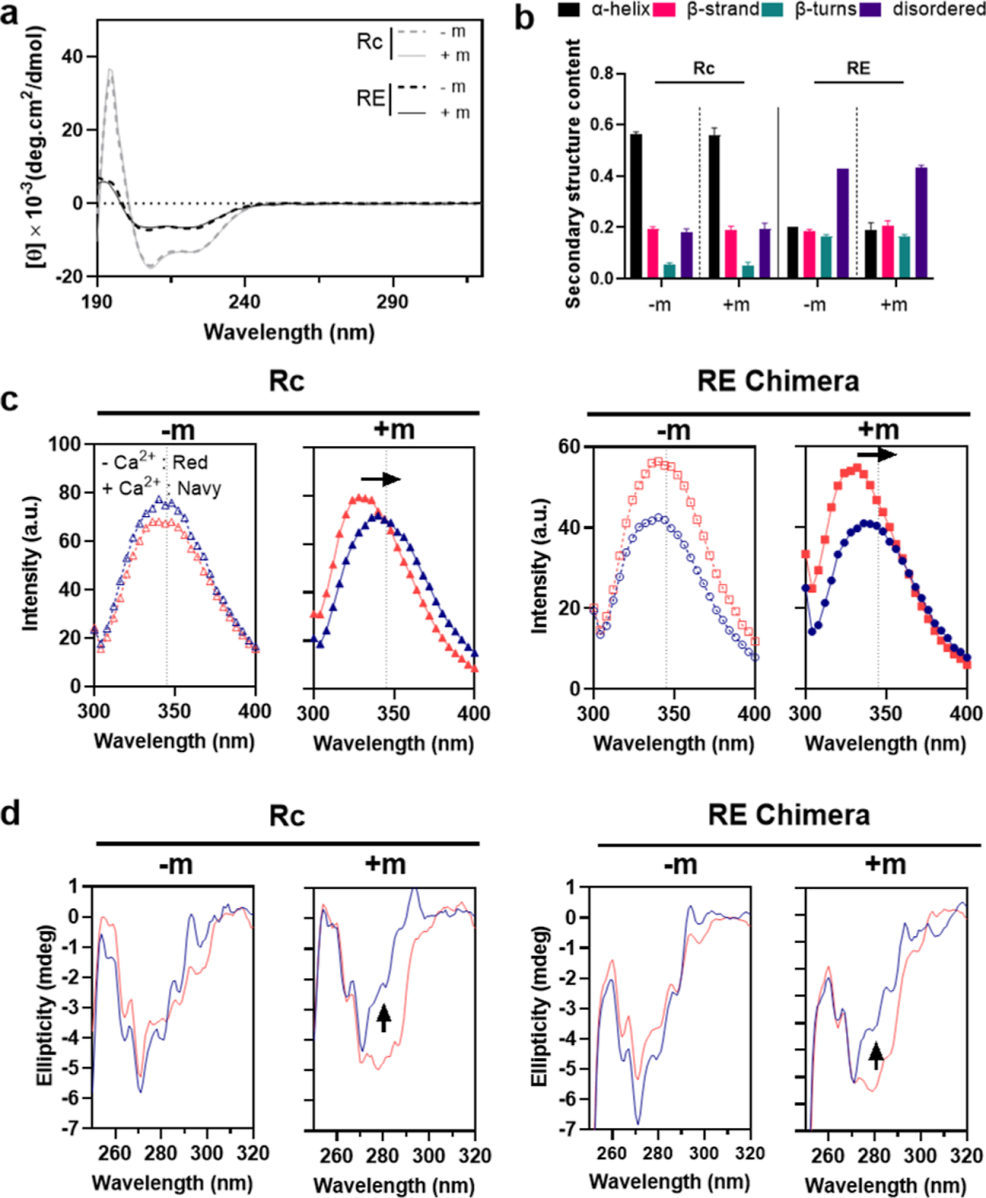
Structural and functional integrity of recoverin in RE fusions. (a,b) Far-UV CD spectra reveal an α-helical structure in recoverin (Rc) and a mixture of α-helical (from R-domain) and random coil (from E-domain) structures in RE in apo form, unaffected by myristoylation (±m). Error bars represent standard deviations from two replicates. (c) Tryptophan fluorescence is red-shifted in both Rc and RE, observed only with myristoylation (+m) and calcium (+Ca^2+^), consistent with the displacement of the lipid from recoverin’s core. The dashed line at 345 nm serves as a visual guide. (d) Near-UV CD spectra of myristoylated Rc and RE demonstrate a decrease in signal intensity at 280 nm when calcium is added (+m, –Ca^2+^ → +Ca^2+^), reflecting a reduction of asymmetry in the environment of the tryptophan residues. This suggests that comparable tertiary structural changes occur in Rc and in the R-domain of the fusion.

To examine the structural and functional integrity of recoverin with the RE fusion, we compared its biophysical properties with Rc using circular dichroism (CD) and tryptophan autofluorescence. Far-UV CD showed that the apo RE combines the R-domain’s α-helical structure^59^ with random coil and β-turn structures of the ELP-domain,^60^ a composition reflective of each domain’s size and unaffected by myristoylation (±m) (Figure 2a,b). To confirm that the recoverin is functional—i.e., that exposure to calcium triggers conformational change that drives release of the myristoyl—we utilized tryptophan intrinsic fluorescence (Figure 2c) and near-UV CD spectroscopy (Figure 2d). We selected these assays because the secondary structure of the recoverin does not change significantly upon binding to calcium, resulting in modest changes in the far-UV CD spectra. On the other hand, one of the recoverin’s three tryptophan residues (W31) forms a hydrophobic pocket that sequesters the lipid in the absence of Ca^2+^. Calcium binding leads to the lipid’s exposure, causing a red shift in fluorescence.^61^ Our experiments demonstrated this shift in autofluorescence for both Rc and RE, but crucially only when the myristoyl group and calcium were both present (Figure 2c), indicating functional calcium binding and consistent conformational changes. The emission spectra of Rc and RE in the absence and presence of calcium show a minor blue shift for Rc from 342 to 340 nm without the lipid. However, lipidated Rc and RE exhibited a significant red shift upon calcium binding, 331 to 341 and 329 to 337 nm, respectively (horizontal arrows in Figure 2c). Intrinsic fluorescence results were complemented by near-UV CD spectroscopy, which is sensitive to tertiary structure changes.^62^ Consistent with prior studies,^63^ the recoverin near-UV spectrum showed negligible change upon calcium binding without lipid (Figure 2d). However, lipidated recoverin displayed a decrease in the signal intensity at 280 nm in the presence of calcium, indicating a reduction in the asymmetry around tryptophan residues. Similar spectral changes in RE fusion suggest that the R-domain undergoes comparable tertiary structural adjustments (vertical arrows in Figure 2d). These results confirm that the R-domain maintains its secondary structure and calcium responsiveness, a prerequisite for modulating the temperature-responsive assembly of the E-domain.

### 3.2 RE Fusion Exhibits Distinct Thermal Stability and Thermal Transitions in Myristoylation- and Calcium-Dependent Manner

**Figure 3:**
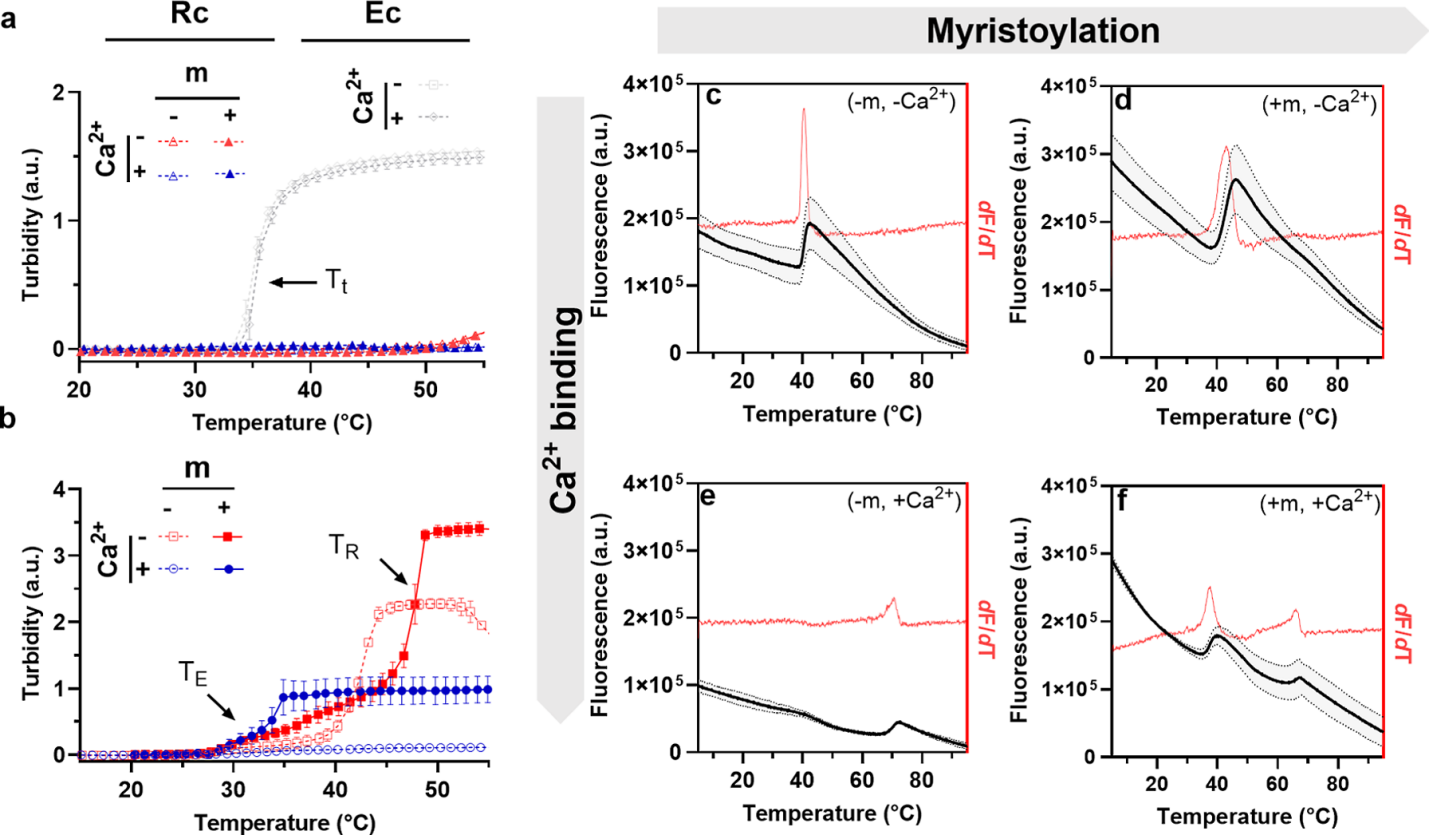
Myristoylation- and calcium-dependent variations in the temperature-responsive phase behavior of RE fusions. (a,b) Variable-temperature turbidimetry shows the effects of myristoylation (±m) and calcium (±Ca^2+^) on the phase behavior of both constituent domains and RE fusions. (a) The R-control is thermally stable up to 55 °C, while the E-control exhibits a distinct, calcium-independent phase transition. (b) Fusion constructs display unique behaviors, including single or dual phase transitions, modulated by myristoylation and calcium. Error bars indicate standard deviations from duplicate measurements. (c–f) Thermal shift assays monitor temperature-triggered changes in the R-domain of the chimera using SYPRO Orange dye, which preferentially binds to exposed hydrophobic regions of the R-domain. (c) In the absence of myristoylation and calcium (−m, −Ca^2+^), (d) with myristoylation alone (+m, −Ca^2+^), (e) with calcium alone (−m, +Ca^2+^), and (f) with both myristoylation and calcium (+m, +Ca^2+^). Myristoylation and particularly calcium binding increase the melting temperature of the R-domain. With both modifiers present, the RE chimera exhibits two distinct thermal transitions at 37 and 66 °C, attributed to different structural changes within the R-domain: the lower transition suggests an emergent structural rearrangement, while the higher temperature corresponds to the melting point of the R-domain. Dashed lines and shaded areas indicate the standard deviation from three measurements. The red line in each panel represents the first derivative of the averaged curve.

To examine how the R-domain influences the E-domain’s temperature-dependent phase separation in the presence and absence of myristoylation (±m) and/or calcium (±Ca^2+^), we used variable-temperature turbidimetry. The RE fusion protein demonstrated distinct phase separation behavior from its constituent domains, which is sensitive to both calcium (±Ca^2+^) and myristoylation (±m) (Figure 3a,b). The turbidity of Rc (±m) did not increase up to 50 °C independent of calcium presence. A slight increase in turbidity was only observed near 55 °C in the absence of Ca^2+^, indicating that the recoverin is thermally stable and does not undergo phase separation under these conditions (Figure 3a).^64^ In contrast, the turbidity of Ec solution sharply increased at temperatures above a critical temperature, with this transition temperature (*T*_t_) being independent of calcium, as evidenced by the overlapping turbidity profiles of ± Ca^2+^ samples (Figure 3a). In the RE fusion, however, the phase-separation behavior varied significantly with the presence of calcium and myristoylation (Figure 3b). Without calcium, the turbidity plots showed two inflection points, likely indicative of independent thermal transitions in the E-domain (*T*_E_) and R-domain (*T*_R_). However, in the presence of calcium, only one inflection point appeared as the turbidity plateaued at higher temperatures, particularly notable in RE (+m, +Ca^2+^). This intriguing variation prompted further investigations.

We first used a thermal shift assay to determine the origins of each transition, followed by concentration-dependent turbidimetry to explore how the fusion of R- and E-domains affects each other and the roles of myristoylation and calcium binding in modulating these interactions. To identify the specific transitions associated with each protein domain, we conducted a thermal shift assay by incubating RE (±m, ±Ca^2+^) with SYPRO Orange (Figure 3c–f). Given that SYPRO Orange fluorescence increased with the unfolding of Rc (Figure S4) but not during the phase separation of Ec (Figure S5), SYPRO Orange fluorescence can be used to specifically monitor transitions in the R-domain within the RE fusion. Compared to Rc, thermal stability of the R-domain is reduced in the RE fusion protein [47.5 °C in R_c_ (−m, −Ca^2+^) vs 40.4 °C in RE (−m, −Ca^2+^)], likely due to the fusion of a large disordered E-domain, but is enhanced both by myristoylation [43.2 °C in RE(+m, −Ca^2+^)] and calcium binding [66.3 °C in RE (−m, + Ca^2+^)] (Figure 3c–e and Table S2). When both myristoylation and calcium are present, we observed two distinct transitions at 37.5 and 66.3 °C in RE(+m, +Ca^2+^) (Figure 3f). These transitions, which we observed only in the RE fusion, suggest that the R-domain undergoes a two-stage conformational change under these conditions. Notably, the lower temperature closely aligns with the point at which the turbidity plots for this construct begin to plateau and, as discussed later in the paper, corresponds to drastic changes in the nanoassembly of RE under these conditions.

### 3.3 Recoverin Alters the Phase Behavior of ELP in the RE Fusion

To elucidate the influence of the R-domain on the E-domain phase behavior, we utilized concentration-dependent turbidimetry. Unlike globular proteins, whose melting temperatures show minimal concentration dependence due to primarily intramolecular interactions, the transition temperature of ELP significantly varies with concentration, reflecting both inter- and intramolecular aggregation during phase separation.^65^ Previous studies have established an empirical linear relationship between the transition temperature of ELP and the natural logarithm of its concentration, with changes in the intercept and slope indicating alterations in the ELP’s hydrophobicity or quaternary organization.^66^ Our data showed that fusion with the R-domain and myristoylation (but not calcium alone) significantly reduced both the intercept and slope of these plots for the E-domain (Figure S6 and Table S3). This alteration suggests an increase in the hydrophobicity of the E-domain within the RE fusion and implies that myristoylation may influence the quaternary structure and assembly of the fusion protein. Our results are in agreement with a model whereby RE fusion undergoes two temperature-dependent transitions, one that reflects the ELP-domain’s lower-critical solubility temperature (*T*_E_) and the other (*T*_R_) that reflects thermal unfolding of the R-domain. Although *T*_E_ was seen to not depend on calcium, *T*_R_ displayed strong myristoylation and even stronger calcium dependence. Additionally, as mentioned earlier, the R-domain demonstrates a unique two-stage conformational change, suggesting intricate interactions between these factors that specifically influence the behavior of the RE fusion. These findings highlight the roles of myristoylation and calcium in modulating RE’s phase behavior, thus supporting our hypothesis that liposwitching (calcium-dependent exposure of the myristoyl group) can effectively control the fusion’s phase separation. This is particularly significant because, while the phase behavior of ELP-fusions can be tuned by altering their composition,^67^ such adjustments typically require genetic modifications and re-expression of the protein. In contrast, our results show that the R-domain’s regulatory control over the E-domain’s phase behavior can be modulated by two distinct factors—myristoylation and calcium—without any need to alter the E-domain’s sequence.

### 3.4 RE Fusion Exhibits Myristoylation- and Calcium-Dependent Nanoassembly

**Figure 4:**
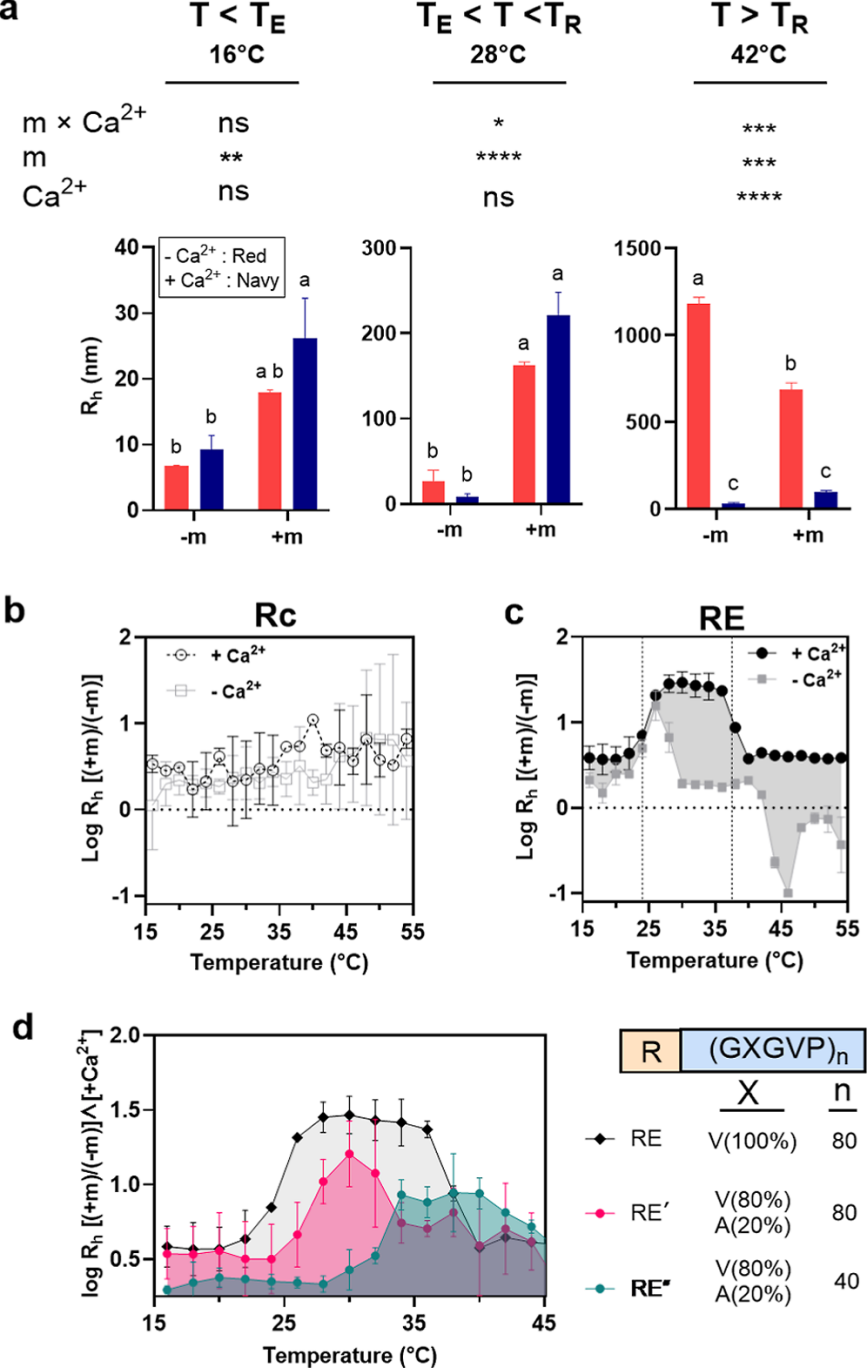
Emergent nanoassembly of RE fusions is influenced by the interaction between myristoylation (±m) and calcium (±Ca^2+^). (a) Variable-temperature dynamic light scattering (DLS) elucidates that nanoassembly of RE fusions is regulated by both myristoylation and calcium, and their interaction is statistically significant above the transition temperature (*T*_E_). Error bars represent standard deviations from two independent samples, each measured in triplicate. In each temperature regime, compact display letters are used for multiple comparisons; groups that do not share the same letter differ significantly, as determined by the Tukey test at the 5% significance level. Statistical significance of factors is indicated by p-values: ns (*p* > 0.5), * (*p* < 0.05), ** (*p* < 0.01), *** (*p* < 0.001), **** (*p* < 0.0001). For detailed ANOVA results, see Table S4. (b,c) Transformed DLS results highlight the temperature-dependent interactions of myristoylation and calcium in the R-control (b) and RE chimera (c). The DLS data confirm that the liposwitching thresholds align closely with TE and TR (indicated by vertical dashed lines), supporting the hypothesis that calcium-dependent exposure of the myristoyl group modulates E-domain aggregation. (d) Modifying the composition of the E-domain by increasing its hydrophilicity (*E*′) and decreasing its length (*E*″) can be used to modulate the liposwitching threshold.

After confirming that recoverin’s myristoyl switch alters the RE fusion’s phase behavior, we utilized dynamic light scattering (DLS) to assess its impact on nanoassembly across various temperatures. The DLS results revealed that myristoylation and calcium binding modulate nanoassembly as well as complex temperature-dependent interactions between these factors (Figures 4a and S7 and Table S4). For example, at *T* < *T*_E_, myristoylation alone significantly increased aggregate sizes, whereas calcium binding and the interaction between myristoylation and calcium (mxCa^2+^) were not statistically significant. This suggests that myristoylation alters the balance of the amphiphilicity of the hydrated E-domain and R-domain sufficiently to drive nanoassembly. However, at *T*_E_ < *T* < *T*_R_, just as the ELP domain is starting to dehydrate, both myristoylation and its interaction with calcium critically influence the aggregate sizes. At *T* > *T*_R_, all factors (myristoylation, calcium, and their interaction) significantly dictate the assembly size. This complex interplay led us to adopt a different approach to analyze the variable-temperature DLS results.

We analyzed the interplay between myristoylation and calcium by plotting the natural log ratio of hydrodynamic sizes, Log *R*_h_ [(+m)/(−m)], across temperatures with and without calcium (±Ca^2+^), Figure 4b,c. Positive values indicate larger sizes for myristoylated constructs compared to nonmyristoylated ones. Deviations from zero on each curve reflect the impact of myristoylation, while differences between the ±Ca^2+^ curves reveal their combined effect. This analysis demonstrates that (1) the RE fusion exhibits unique, temperature-dependent behavioral patterns (Figure 4c) absent in the recoverin single-domain control (Figure 4b); and (2) the temperature-dependent interactions between myristoylation and calcium are particularly pronounced at *T* > *T*_E_. These findings underscore that calcium-induced changes in the R-domain have a maximal effect on the E-domain’s phase behavior when recoverin is myristoylated, supporting the concept of “liposwitching” as a mechanism for dynamic regulation of IDP-driven aggregation.

In the absence of calcium, myristoylation alone has a minimal impact on the temperature-dependent aggregation of RE, with the Log *R*_h_ [(+m)/(−m)] ratio staying close to zero across most temperatures (a behavior also observed for Rc, which lacks the E-domain). Minor deviations at 24–28 and 44–48 °C in Figure 4c are observed because myristoylation lowers *T*_E_ and increases *T*_R_. As the temperature approaches these transition points, the construct with the lower threshold initially increases in size, but this increase is offset once the temperature surpasses the corresponding transition point for the other construct, explaining the sharp peaks in the −Ca^2+^ plot (gray rectangles) in Figure 4c.

The divergence between the −Ca^2+^ and +Ca^2+^ curves in Figure 4c (shaded gray region) vividly illustrates three distinct regimes of temperature-dependent interplay between myristoylation and calcium (denoted with vertical dashed lines). In the first regime, at *T* < *T*_E_, the curves do not diverge, indicating that the liposwitching effect requires IDP-driven aggregation to manifest. In the second regime, where *T*_E_ < *T* < *T*_R_, the curves show maximal divergence, underscoring the role of myristoylation and calcium in regulating the IDP-driven nanoassembly. Finally, at *T* > *T*_R_—corresponding to the lower transition observed for RE(+m, +Ca^2+^)—the differences between the curves are reduced but still evident. This reduction is attributed to a change in the assembly of RE (+m, +Ca^2+^) samples around 35 °C, coinciding with *T*_R_ observed in thermal shift assays and marked by the formation of smaller assemblies. Above this temperature, calcium-bound recoverin stabilizes the aggregates, resulting in leveled transformed DLS plots, while noncalcium-bound samples undergo recoverin denaturation, losing their influence on the E-domain’s properties. The close alignment of nanoassembly with *T*_E_ and *T*_R_ reinforces our hypothesis that calcium-dependent exposure of the myristoyl group in the R-domain effectively regulates the E-domain’s temperature-triggered aggregation in the RE fusion.

### 3.5 Modulating RE Fusion Liposwitching Threshold by Changing Composition of the E-Domain

Based on the observed dependence of RE nanoassembly on the interaction between myristoylation and calcium that occurs above *T*_E_, we hypothesized that this behavior can be further modulated by altering the E-domain’s composition. To investigate this, we engineered two additional variants, RE′, which displays increased hydrophilicity by substituting 20% of the valines with alanine, and RE″, which combines increased hydrophilicity with decreased length of the E-domain (from 80 to 40 pentapeptide repeats). Both modifications are anticipated to raise the transition temperature of the ELP domain,^68^ leading to increased *T*_E_ values for RE′ and RE″, following the trend *T*_E_ (RE) < *T*_E_ (RE′) < *T*_E_ (RE″).The transformed DLS results, shown in Figures 4d and S8, confirmed that these compositional changes resulted in changes to the liposwitching threshold, as evidenced by the shifts in the plots to higher temperatures. This outcome is significant because it demonstrates the feasibility of manipulating the responsiveness to calcium-triggered lipid exposure by strategically modifying the phase boundaries of the disordered domain. Consequently, we anticipate that this strategy could be extended to other intrinsically disordered proteins that undergo phase separation under isothermal conditions by adjusting factors such as pH, ionic strength, or specific molecular interactions.

### 3.6 RE Fusion Assembles into Topologically Distinct Nanostructures Depending on the Temperature, Myristoylation, and Calcium

**Figure 5:**
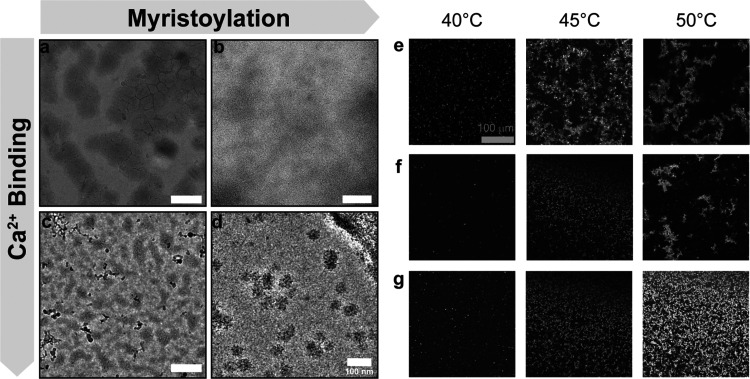
Nanoassembly of RE fusion constructs. (a–d) Cryo-TEM at 35 °C visualizes the nanoscale organization of RE assemblies under different conditions: (a) without myristoylation or calcium (−m, −Ca^2+^); (b) with myristoylation alone (+m, −Ca^2+^); (c) with calcium alone (−m, +Ca^2+^); and (d) with both myristoylation and calcium (+m, +Ca^2+^). (e–g) Confocal microscopy is used to visualize the mesoscale organization of RE assemblies at 40–50 °C: (e) without myristoylation or calcium (−m, −Ca^2+^); (f) with myristoylation alone (+m, −Ca^2+^); and (g) with both myristoylation and calcium (+m, +Ca^2+^).

Building on the DLS analysis, we used cryo-TEM to explore how myristoylation and calcium affect the nanoscale organization of RE assemblies (Figure 5a–d), particularly at temperatures above *T*_E_, where the increased hydrophobicity of the E-domain significantly drives the differences between the constructs. Representative cryo-TEM images obtained by vitrification of samples incubated at 35 °C illustrate how myristoylation (±m) and calcium binding (±Ca^2+^) modulate the assembly of the fusion protein. Calcium notably influences both nanoscale clustering and the higher-order organization of clusters, while the effect of myristoylation is subtler and dependent on the presence of calcium, highlighting the complex interplay between these two factors. In the absence of myristoylation, RE fusions form a network of interconnected mesoglobules when calcium is absent (Figure 5a) and worm-like micelles when calcium is present (Figure 5c). More specifically, myristoylation alone [RE (+m, −Ca^2+^)] alters mesoglobular formation, leading to more diffuse structures, which we propose is due to altered hydration of the R-domain and E-domain postmyristoylation, even when the myristoyl group is buried within the hydrophobic core of the R-domain. This hypothesis aligns with our concentration-dependent turbidimetry data (Figure S6), suggesting that myristoylation brings the physicochemical properties of the R- and E-domains closer, resulting in less distinct amphiphilic characteristics that control RE assembly. When both myristoylation and calcium are present (+m, +Ca^2+^), RE forms densely packed clusters, highlighting the significant impact of these combined factors on the system’s assembly (Figure 5d). This transformation demonstrates the profound combined effects of lipidation and calcium on the system’s assembly, confirming that the first transition observed in the thermal shift assay of RE (+m, +Ca^2+^) (Figure 3d) coincides with significant organizational changes.

To examine mesoscale assemblies of RE proteins, we used confocal scanning laser microscopy (CSLM) at temperatures ranging from 40 to 50 °C (Figure 5e–g), where cryo-TEM was impractical due to vitrification challenges at higher temperatures. CSLM analysis revealed that calcium significantly limits the extent of aggregation, while lipidation subtly modifies the pattern and distribution of RE assemblies. Specifically, RE (−m, +Ca^2+^) assemblies remained below the resolution of CSLM even when heated to 50 °C (Figure S9). In contrast, the other conditions [RE (−m, −Ca^2+^) and RE (+m, ±Ca^2+^)] produced larger structures that adhered to the microscopy slide chamber, making them observable at these temperatures. In the absence of both myristoyl and calcium, RE formed large fractal clusters at 45 and 50 °C (*T* > *T*_R_) (Figure 5e). The morphology of these clusters was noticeably different from the spherical coacervates formed by Ec (Figure S10). Conversely, myristoylation alone [RE (+m, −Ca^2+^)] delayed aggregate formation until 45 °C due to an elevated transition temperature (TR) of the R-domain and resulted in less interconnected aggregates at higher temperatures compared to its nonmyristoylated counterpart (Figure 5f). Interestingly, when both myristoyl and calcium were present [RE (+m, +Ca^2+^)], the resulting structures differed markedly, displaying densely packed arrays of smaller aggregates across the surface of the chamber. Although these structures were larger than those observed in cryo-TEM, suggesting that nanoclusters of RE (+m, +Ca^2+^) may interact to form higher-order assemblies, they do not aggregate as extensively as under calcium-free conditions. We are cognizant that these higher-order assemblies may also be influenced by surface adherence. Regardless, these data clearly demonstrate that liposwitching can be used to alter nano- and mesoscale assemblies, highlighting its potential as a mechanism for controlling the assembly without altering the primary sequence.

## 4 Conclusions

In summary, we designed a fusion protein that successfully combines the natural calcium-dependent myristoyl switch of recoverin with phase separation of an IDP. We demonstrated that the fusion’s phase behavior and nanoclustering can be effectively regulated by two distinct signals, myristoylation (i.e., lipidation) and calcium. The E-domain used in our study serves as a model IDP due to its well-characterized temperature-triggered phase-separation properties. Here, we showed that these properties and the IDP-driven nanoassembly can be controlled by additional factors, such as calcium and the lipidation status of the fusion partner. Moreover, we present evidence that this process can be further modulated by altering the E-domain composition. The sequence-programmable thermoresponsiveness of ELPs has been used to engineer stimuli-responsive biopolymers and nanomaterials for drug delivery and tissue engineering. This makes RE fusions particularly useful in biomedical settings where localized thermal control is possible, facilitating targeted therapeutic delivery.^69−71^

Overall, the principles of integrating responsiveness to multiple factors into an IDP-driven nanoassembly are anticipated to extend to other IDPs and myristoyl switches. For example, the phase separation of the IDP can also be modulated by pH, the use of light-responsive amino acids, or ligand binding, making the system versatile for environments where temperature control is impractical.^72^ Additionally, by exploring other myristoyl switches and myristic acid analogues,^73^ such as the unsaturated fatty acids found in recoverin isoforms,^74^ we can fine-tune the response of recoverin fusions and develop nanobiomaterials responsive to a diverse range of physiological triggers like pH and membrane composition.^75−77^

This in vitro study represents a foundational step in understanding the intricate interplay among lipidation, the calcium-dependent allosteric switch, and the IDP-driven assembly. The rigorous biophysical and soft-matter characterizations performed here are essential for refining and advance this emerging class of biomaterials. Future work will focus on integrating these liposwitch systems into more complex biological environments, aiming to develop programmable materials that can respond to and influence cellular processes in real-time.^78^ Envisioning a future that mirrors the adaptability of riboswitches,^79^ these liposwitch-based platforms could dynamically interact with and modulate cellular signals, opening new frontiers in synthetic biology and cellular engineering.

## Data Availability

The data supporting this article have been included as part of the Supporting Information.

## Supporting Information Available

Detailed materials and methods including cloning, protein expression, purification, RP-HPLC, fluorescent labeling, SDS–PAGE, MALDI–TOF–MS, and LC–MS analysis; DNA and protein sequences; theoretical molecular weight and observed m/z for various constructs in this study; transition temperatures observed in the thermal shift assay for R-control and RE chimera; concentration dependencies of transitions observed in VT-turbidimetry data; ANOVA results; SDS-PAGE characterization; LC–MS results; thermal shift assay results; untransformed variable-temperature DLS data; and visualization of RE and ELP using confocal microscopy (PDF)
