# Supplementary material for: Genetically Engineered Liposwitch-Based Nanomaterials
Source: Biomacromolecules. 2024 Nov 4;25(12):8058–68. doi: 10.1021/acs.biomac.4c01388 (PMC11632658; doi:10.1021/acs.biomac.4c01388)
Supplement: Supplementary file 1 — bm4c01388_si_001.pdf [file bm4c01388_si_001.pdf]

Genetically Engineered Liposwitch-based Nanomaterials

Md. Shahadat Hossain, Alex Wang, Salma Anika, Zhe Zhang, Davoud Mozhdehi*

*Corresponding author's email: dmozhdeh@syr.edu

Table of Contents

1. Materials S1
2. Cloning S2
3. Protein Expression S2
4. Protein purification S2
5. RP-HPLC S3
6. Fluorescent Labeling S3
7. Sodium dodecyl-sulfate polyacrylamide gel electrophoresis S3
8. MALDI-TOF-MS S3
9. LC-MS Analysis of Trypsin Digest S3
10. DNA and Protein sequences S4
Recoverin DNA sequence S4
Sequence of proteins S4
11. Supplementary Tables S5
12. Supplementary Figures S7
13. References S16

1. Materials

Restriction enzymes, ligase, NEBuilder ® HiFi DNA Assembly cloning kit, Monarch ® DNA Gel Extraction Kits, corresponding buffers, and chemically competent Eb5alpha and BL21(DE3) cells were purchased from New England Biolabs (Ipswich, MA). DNA oligonucleotides and gene fragments were synthesized by Integrated DNA Technologies (Coralville, Iowa). PureLink™ Quick Plasmid Mini-Prep Kits, high-performance liquid chromatography-(HPLC) grade acetonitrile, liquid chromatography mass spectrometry (LC-MS) grade water, acetonitrile, and formic acid, HisPur™ Cobalt Resin Spin Columns, Zeba™ desalting columns (3k MWCO), sinaptic acid, AlexaFluor-488 NHS Ester, mass spectroscopy grade Pierce™ trypsin protease, myristic acid, agarose, tris-HCl, imidazole, sodium chloride, ethylene glycol-bis(β-aminoethyl ether)-N,N,N′,N′-tetraacetic acid (EGTA), Calcium Chloride ($CaCl_2$+), ammonium bicarbonate, sodium bicarbonate, chloramphenicol, ampicillin sodium salt, and anhydrous DMSO was purchased from Thermo Fisher Scientific (Rockford, IL). Mini-PROTEAN® TGX Stain-Free™ Precast Gels, Precision Plus Protein™ Unstained Protein Standards, and 4x Laemmli buffer were purchased from BioRad Laboratories, Inc (Hercules, CA). Polyethersulfone (PES, 0.22 μm) syringe filters, ZipTip™ Pipette filters, Amicon® Ultra-15 Centrifugal Filter (3k MWCO), ProteoMass™ Albumin and Aldolase MALDI-MS standard, sinaptic acid, ammonium bicarbonate, and trifluoroacetic acid (TFA), phenylmethylsulfonyl fluoride (PMSF) were purchased from Sigma-Aldrich (St. Louis, MO). Isopropyl β-D-1-thiogalactopyranoside (IPTG) was purchased from GoldBio (St. Louis, MO). Kanamycin sulfate, sodium chloride, yeast extract, and tryptone was purchased from Bio Basic (Markham, ON, CA). μ-Slide 8 Well Glass Bottom microscope slide was purchased from Abidi. The carbon-coated grid (CF300-Cu) was purchased from Electron Microscopy Sciences (Hatfield, PA). Deionized water was obtained from a Milli-Q® system (Millipore SAS, France). Simply Blue™ SafeStain was purchased from Novex (Carlsbad, CA). All chemicals were used as received without further purification.

2. Cloning

The copy DNA (cDNA) for *Bos taurus* recoverin (Uniport Accession # P21457) was obtained using Sanger sequencing from a template plasmid kindly provided by Dr. James Ames (University of California, Davis). Modifications were made to the cDNA sequence to remove internal restriction enzyme sites necessary for assembly with the ELP gene, including *AcuI*, *BseRI*, and *BglI*. These modifications were achieved through silent mutations and the addition of 20–40 base pair homology regions compatible with the recipient vector. The modified gene fragment was synthesized by IDT DNA Technologies and subsequently cloned into a linearized pET-24 plasmid using the NEBuilder® HiFi DNA Assembly according to manufacturer protocol.

The fusion protein's (RE) gene was synthesized through two rounds of "plasmid reconstruction by recursive directional ligation (PRe-RDL)" technique.[1] First, plasmids carrying the recoverin and ELP genes underwent double digestion with BglI, and either AcuI or BseRI. Following this, DNA fragments containing either Rec or ELP genes were separated via agarose gel electrophoresis and then purified using the Monarch gel extraction kit, according to the manufacturer's protocol. These purified fragments were ligated together using the Quick Ligase Kit (New England Biolabs). The ligated products were subsequently introduced into chemically competent NEB5a cells, and colonies were selected based on kanamycin resistance. Following DNA sequencing, this procedure was replicated to incorporate the C-terminal octa-histidine tag (8x-His). Finally, nanopore sequencing was utilized to confirm the sequence of all the plasmids.

3. Protein Expression

Protein expression was conducted using *E. coli* BL21(DE3) strain in 2x yeast extract and tryptone (2xYT) broth. For constructs requiring myristoylation, an orthogonal plasmid encoding yeast N-myristoytransferase (NMT) was co-transformed.[2] This plasmid contained a p15A origin and included a chloramphenicol resistance marker. To accommodate the secondary plasmid, growth media for expression of lipidated constructs was supplemented with both kanamycin (45 µg/mL) and chloramphenicol (25 µg/mL). On the other hand, the unmodified proteins were using a single plasmid in 2xYT supplemented with only kanamycin (45 µg/mL).
A freshly transformed colony was used to inoculate a 50 mL seed culture, which was incubated at 37 °C with shaking at 250 rpm until the culture achieved the optical density (OD_{600}) of 0.6. The seed culture was used to inoculate 1-liter expression cultures, using a 1:100 inoculum ratio. These expression cultures were cultivated at 37 °C with shaking until they reached an optical density OD_{600} of 0.6-0.8. Subsequently, the temperature of the incubator was lowered to 28 °C. For the myristoylated constructs, 100 µM myristic acid (prepared as 1000x concentrated solution in DMSO) was added to the expression media. After an incubation period of 15 minutes, protein expression was induced by supplementing the media with 0.5 mM IPTG. Eighteen hours post-induction, the cells were harvested using centrifugation (5000 x*g*, 20 min, 4 °C). The cell pellets were then resuspended in 5 mL of Tris-buffered saline (TBS; 50 mM Tris-HCl, 137 mM NaCl, pH 7.4) per liter of expression media, and subsequently stored at -80 °C before purification.

4. Protein purification

Protein purification was conducted in two stages: initial purification using immobilized metal affinity chromatography (IMAC) and subsequent polishing with reverse-phase high-performance liquid chromatography (RP-HPLC).[3,4] Before cell lysis, the frozen suspensions were thawed and treated with 1 mM phenylmethylsulfonyl fluoride (PMSF). Lysis was conducted on ice using sonication (Fisher, Model 505 Sonic Dismembrator), consisting of two cycles (1:30 min each, 10 s on, 60 s off, 60W), followed by clarification through centrifugation (21000 x*g*, 10 min, 4 °C). The cell lysate was supplemented with 10 mM Imidazole and incubated with HisPur^{TM} Cobalt resin at a ratio of 10 mL lysate per 3 mL resin for 30 minutes. The resin was subsequently washed three times with two volumes of wash buffer (50 mM Tris-HCl, 10 mM imidazole, 300 mM NaCl, pH 7.4). His-tagged proteins were eluted by incubating the resin five times with one volume of elution buffer (50 mM Tris-HCl, 300 mM imidazole, 300 mM NaCl, pH 7.4). The purity of the elution fractions were confirmed by SDS-PAGE before combining. The pooled fractions were concentrated using a centrifugal filter unit (Amicon® Ultra-15, 3k MWCO), following the manufacturer's instructions. The concentrated eluates were further purified to homogeneity (>95% purity) using preparative reverse-phase HPLC (RP-HPLC), with details provided below. The HPLC fractions were flash-frozen in liquid nitrogen, lyophilized, and the resulting protein powder was stored at -20 °C.

The ELP control (Ec) was purified by Inverse Transition Cycling (ITC) according to previously published protocols.[5] Briefly, ITC utilized the lower-critical solubility temperature (LCST) property of the ELP, wherein the protein is soluble at low temperatures and insoluble at high temperatures. Through isothermal switching, the ELP can be separated from protein contaminants. Lysis was performed via sonication and the lysate was clarified by centrifugation (parameters above). Protein contaminants sensitive to high temperatures were removed through a "bakeout," where the lysis supernatant was heated at 50 °C for 20 minutes, cooled on ice for 10 minutes, and clarified by centrifugation. The ELP was precipitated from solution by adding crystalline NaCl and was collected by centrifugation. The resulting ELP pellet was resuspended in cold ddH_2O and centrifuged at 4 °C to remove any final contaminants. Purity was assessed through analytical RP-HPLC

5. RP-HPLC

HPLC analysis was performed using Shimadzu Prominence HPLC systems, with an LC2030 i-series for analytical and a Prominence modular HPLC for preparative scales. Flow rates were maintained at 1 mL/min for analytical HPLC and 4.2 mL/min for preparative HPLC. Both systems employed Phenomenex Jupiter C18 columns (5 µm, 300 Å), 250 x 4.6 mm for analytical and 250 x 10 mm for preparative applications. The mobile phase consisted of a linear gradient, starting with solvent A (H_2O + 0.1% TFA) and transitioning to 90% solvent B (Acetonitrile + 0.1% TFA) and 10% solvent A over 40 minutes. Background correction of HPLC traces was achieved by subtracting a blank trace acquired under identical conditions.

6. Fluorescent Labeling

Lyophilized proteins were reconstituted at 1 mg/mL in 100 mM sodium carbonate buffer (pH 9), with stirring at 4 °C. Alexa Fluor™ 488 NHS Ester dye was prepared in anhydrous DMSO at 0.5 mM concentration. To the protein solution, 30 nmol of the dye stock was added and mixed under stirring for 3 hours at 4 °C. The reaction mixture was subsequently concentrated 4-fold using an Amicon® ultra-15 centrifugal filter (3k MWCO). Excess dye was removed by passing the concentrated mixture through a Zeba™ desalting column (Thermo Fisher Scientific). The efficiency of labeling was determined by measuring the fluorophore concentration at 494 nm (ε = 71000 $M^{-1}.cm^{-1}$) and the protein concentration at 280 nm (ε = 25440 $M^{-1}.cm^{-1}$).

7. Sodium dodecyl-sulfate polyacrylamide gel electrophoresis

The purity and molecular weight of the purified proteins were assessed using 4-20% Mini-PROTEAN® TGX Stain-Free™ Precast Gels. Initially, the gels were visualized under UV light with a BioRad Gel Doc EZ Imager, followed by staining with SimplyBlue™ (Coomassie Blue) to detect any residual protein contaminants. All samples for protein gel analysis were prepared following the Laemmli method.

8. MALDI-TOF-MS

Matrix-assisted laser desorption/ionization time-of-flight mass spectrometry (MALDI-TOF-MS) was conducted on a Bruker microflex LRF with a microScout ion source. Samples for analysis were prepared by mixing an equal volume of sample and synaptic acid (SA) matrix (SA was prepared at a concentration of 10 mg/mL in 70% acetonitrile + 0.1% TFA). 2 µL of this mixture was spotted on a steel target plate. All spectra were calibrated against an aldolase (39,211.28 Da) and albumin standard (66,429.09 Da).

9. LC-MS Analysis of Trypsin Digest

For LC-MS analysis, a 2.5 µL aliquot of the protein solution (2 mg/mL) was mixed with 100 mM ammonium bicarbonate (pH 8). The mixture underwent trypsin digestion (1:50 w/w ratio) for 8 hours at room temperature. The digested sample was desalted using ZipTip® columns. Chromatographic separation was performed using an Ultimate 3000 nano-LC pump (Dionex, Mountain View, CA, USA) and a C18 column (Acclaim PepMap™ 100, 75 µm x 25 cm, nanoViper C18, 3 µm, 100 Å). This system was connected to an LTQ-Orbitrap-ETD XL mass spectrometer (ThermoFisher Scientific, San Jose, CA, USA) equipped with a nanospray ion source (New Objective, Woburn, MA, USA). The loading pump used a mobile phase of 0.1% formic acid in water at 200 µL/min, while the nano-LC pump used 80% acetonitrile with 0.1% formic acid (solvent B). Peptides were eluted at 300 µL/min using a linear gradient from 4% to 55% solvent B over 30 minutes, then ramped from 55% to 90% solvent B over 5 minutes.

10. DNA and Protein sequences

Recoverin DNA sequence

The underlined sequence is the homology area with pET24a plasmid. The recognition sequences of *BseRI* and *BamHI* are highlighted as yellow and green, respectively.

CTAGAAATAATTTTGTTTAACTTTAAGAAGGAGGAGTACATATGGCAACAGCAAGAGTGGGGCCCTGTCCAAGGAGATCCTGGA
AGAGCTGCAGCTGAACACCAAGTTCACGGAGGAAGAGCTGAGCTCCTGGTACCAGTCCTTCCTGAAAGAGTGTCCCAGTGGTC
GGATCACCCGGCAGGAGTTCCAGACCATCTACTCCAAGTTCTTCCCCGAGGCCGACCCCAAGGCCTATGCCCAGCACGTGTTC
CGAAGCTTTGATGCCAACAGCGATGGCACCTTGGACTTCAAGGAGTATGTCATCGCCTTACACATGACCAGCGCGGGCAAGACC
AACCAGAAGCTGGAGTGGGCCTTCTCCCTCTATGACGTGGATGGCAATGGGACCATCAGCAAGAACGAGGTGCTGGAGATTGT
CACGGCTATCTTCAAAATGATCAGCCCTGAGGACACAAAGCATCTCCCAGAAGACGAGAACACTCCGGAAAAGCGAGCAGAGAA
GATCTGGGGATTCTTTGGCAAGAAGGATGATGATAAACTTACAGAGAAAGAATTCATCGAAGGGACCCTGGCCAATAAGGAAATT
CTGCGACTGATTCAATTCGAGCCTCAAAAAGTGAAGGAGAAACTGAAAGAAAAGAAACTCGGCTGATAATGATAATCTTCAGGAT
CCGAATTCGAGCTCCGTCGACAAGCTTGCGGCCGCAC

Sequence of proteins

Recoverin-His **(R)**
GNSKSGALSKEILEELQLNTKFTEEELSSWYQSFLKECPSGRITRQEFQTIYSKFFPEADPKAYAQHVFRSFDANSDGTLDFKEYVIALH
MTSAGKTNQKLEWAFSLYDVDGNGTISKNEVLEIVTAIFKMISPEDTKHLPEDENTPEKRAEKIWGFFGKKDDDKLTEKEFIEGTLANKEI
LRLIQFEPQKVKEKLKEKKLGHHHHHHHHG

Recoverin-ELP(V,80)-His **(RE)**
GNSKSGALSKEILEELQLNTKFTEEELSSWYQSFLKECPSGRITRQEFQTIYSKFFPEADPKAYAQHVFRSFDANSDGTLDFKEYVIALH
MTSAGKTNQKLEWAFSLYDVDGNGTISKNEVLEIVTAIFKMISPEDTKHLPEDENTPEKRAEKIWGFFGKKDDDKLTEKEFIEGTLANKEI
LRLIQFEPQKVKEKLKEKKLGVGVPGVGVPGVGVPGVGVPGVGVPGVGVPGVGVPGVGVPGVGVPGVGVPGVGVPGVGVPGVGVP
GVGVPGVGVPGVGVPGVGVPGVGVPGVGVPGVGVPGVGVPGVGVPGVGVPGVGVPGVGVPGVGVPGVGVPGVGVPGVGVPGV
GVPGVGVPGVGVPGVGVPGVGVPGVGVPGVGVPGVGVPGVGVPGVGVPGVGVPGVGVPGVGVPGVGVPGVGVPGVGVPGVGVP
GVGVPGVGVPGVGVPGVGVPGVGVPGVGVPGVGVPGVGVPGVGVPGVGVPGVGVPGVGVPGVGVPGVGVPGVGVPGVGVPGV
GVPGVGVPGVGVPGVGVPGVGVPGVGVPGVGVPGVGVPGVGVPGVGVPGVGVPGVGVPGVGVPGVGVPGVGVPGVGVPGVGVP
GVGVPGHHHHHHHHG

Recoverin-ELP(V_8/A_2,80)-His **(RE')**
GNSKSGALSKEILEELQLNTKFTEEELSSWYQSFLKECPSGRITRQEFQTIYSKFFPEADPKAYAQHVFRSFDANSDGTLDFKEYVIALH
MTSAGKTNQKLEWAFSLYDVDGNGTISKNEVLEIVTAIFKMISPEDTKHLPEDENTPEKRAEKIWGFFGKKDDDKLTEKEFIEGTLANKEI
LRLIQFEPQKVKEKLKEKKLGVGVPGVGVPGAGVPGVGVPGVGVPGVGVPGVGVPGAGVPGVGVPGVGVPGVGVPGVGVPGAGVP
GVGVPGVGVPGVGVPGVGVPGAGVPGVGVPGVGVPGVGVPGVGVPGAGVPGVGVPGVGVPGVGVPGVGVPGAGVPGVGVPGV
GVPGVGVPGVGVPGAGVPGVGVPGVGVPGVGVPGVGVPGAGVPGVGVPGVGVPGVGVPGVGVPGAGVPGVGVPGVGVPGVGVP
GVGVPGAGVPGVGVPGVGVPGVGVPGVGVPGAGVPGVGVPGVGVPGVGVPGVGVPGAGVPGVGVPGVGVPGVGVPGVGVPGA
GVPGVGVPGVGVPGVGVPGVGVPGAGVPGVGVPGVGVPGVGVPGVGVPGAGVPGVGVPGVGVPGVGVPGVGVPGAGVPGVGVP
GVGVPGHHHHHHHHG

Recoverin-ELP(V_8/A_2,40)-His **(RE")**
GNSKSGALSKEILEELQLNTKFTEEELSSWYQSFLKECPSGRITRQEFQTIYSKFFPEADPKAYAQHVFRSFDANSDGTLDFKEYVIALH
MTSAGKTNQKLEWAFSLYDVDGNGTISKNEVLEIVTAIFKMISPEDTKHLPEDENTPEKRAEKIWGFFGKKDDDKLTEKEFIEGTLANKEI
LRLIQFEPQKVKEKLKEKKLGVGVPGVGVPGAGVPGVGVPGVGVPGVGVPGVGVPGAGVPGVGVPGVGVPGVGVPGVGVPGAGVP
GVGVPGVGVPGVGVPGVGVPGAGVPGVGVPGVGVPGVGVPGVGVPGAGVPGVGVPGVGVPGVGVPGVGVPGAGVPGVGVPGV
GVPGVGVPGVGVPGAGVPGVGVPGVGVPGVGVPGVGVPGAGVPGVGVPGVGVPGVGVPGHHHHHHHHG*

11. Supplementary Tables

Table S1. Theoretical Molecular weight and observed m/z for various constructs in this study

Construct	Theoretical Mw	Observed m/z $[M+H]^+$
R (-m)	24413.46	24384
R (+m)	24,623.46	24601
RE (-m)	57172.31	57239
RE (+m)	57,382.31	57409
	Liposwitch variants	
RE′ (-m)	40568.45	40563
RE′ (+m)	40,778.81	40760
RE″ (-m)	56723.45	56735
RE″ (+m)	56933.81	56916
	Trypsin-digested N-terminal fragment	
GNSK	404.2019	405.2106
m-GNSK	614.4003	615.4094

Table S2. Transitions temperatures observed in the thermal shift assay for R-control and RE-Chimera.[a]

	R-Control		RE-Chimera	
-m, $-Ca^{2+}$	33.0 ± 1.2[b]	47.5 ± 6.3	40.4 ± 0.2	
-m, + Ca^{2+}	76.0 ± 1.9		68.9 ± 1.5	
+m, $-Ca^{2+}$	59.1 ± 4.5		43.2 ± 0.4	
+m, $+Ca^{2+}$	70.5 ± 4.8		37.5 ± 0.1[c]	66.3 ± 0.6

[a] Transitions are defined as the local maximum of the first derivative curve. The data is reported as mean ± std. dev. of three measurements.

[b] This transition appeared as a broad shoulder.

[c] This transition was present in all of the replicates and is unique to RE (+m, $+Ca^{2+}$).

Table S3. Concentration-dependencies of transitions observed in VT-turbidimetry data [a]

Concentration (μM)	ELP		RE							
	- Ca^{2+}	+ Ca^{2+}	-m, -Ca^{2+}		-m, +Ca^{2+}		+m, -Ca^{2+}		+m, +Ca^{2+}	
	T_E	T_E	T_E	T_R	T_E	T_R	T_E	T_R	T_E	T_R
1	39.9 ± 0.2	41.1 ± 1.0	n.d.[b]	45.6 ± 0.2	n.d.[b]	n.d.[c]	29.2 ± 0.7	47.0 ± 0.4	n.d.[b]	n.d.[c]
5	36.7 ± 0.1	36.8 ± 0.4	29.1 ± 0.6	42.7 ± 0.2	n.d. [b]	n.d. [c]	27.3 ± 1.1	46.6 ± 1.5	27.2 ± 0.7	n.d. [c]
10	35.2 ± 0.5	35.3 ± 0.5	26.9 ± 1.1	42.8 ± 0.2	n.d. [b]	n.d. [c]	26.6 ± 0.5	47.4 ± 0.5	26.6 ± 1.4	n.d. [c]
25	32.8 ± 0.0	33.3 ± 0.3	28.1 ± 1.1	41.9 ± 0.0	27.5 ± 2.1	n.d. [c]	25.0 ± 0.6	45.2 ± 1.9	24.3 ± 0.7	n.d. [c]
50	32.0 ± 0.0	31.7 ± 0.1	26.7 ± 1.5	39.7 ± 0.0	26.8 ± 1.4	n.d. [c]	23.5 ± 1.1	n.d. [d]	23.6 ± 0.1	n.d. [c]
75	30.8 ± 0.3	31.4 ± 0.4	24.8 ± 1.1	37.1 ± 0.9	24.6 ± 1.4	n.d. [c]	23.2 ± 0.5	n.d. [d]	22.9 ± 0.2	n.d. [c]
100	30.8 ± 0.4	31.0 ± 0.1	25.7 ± 0.2	n.d.[c]	24.6 ± 0.2	n.d. [c]	24.1 ± 0.8	n.d. [d]	23.7 ± 1.2	n.d. [c]

[a] Transitions are defined as the local maximum of the first derivative curve. The data is reported as mean (°C) ± std. dev. of two measurements.

[b] No transition detectable due to low solution turbidity.

[c] No transition was observed in VT-turbidimetry due to high thermal stability of recoverin.

[d] Second transition not observed due to high sample concentration and detector saturation after the first transition.

Table S4. Analysis of Variance (ANOVA) Results for statistical significance of factors regulating the assembly of RE fusions across three temperature regimes.

Temperature range	Factors	Sum of Squares	dF	Mean Square	F (1, 4)	p-value (summary)
$T < T_E$	Interaction	16.95	1	16.95	1.665	0.2665 (ns)
	Lipid	393.1	1	393.1	38.62	0.0034 (**)
	Calcium	59.64	1	59.64	5.859	0.0727 (ns)
	Residual	40.71	4	10.18		
$T_E < T < T_R$	Interaction	2927	1	2927	12.60	0.0238 (*)
	lipid	60946	1	60946	262.4	P<0.0001 (****)
	calcium	820.5	1	820.5	3.532	0.1334 (ns)
	Residual	929.2	4	232.3		
$T > T_R$	Interaction	156476	1	156476	198.7	0.0001 (***)
	lipid	89700	1	89700	113.9	0.0004 (****)
	calcium	1511655	1	1511655	1920	0.0001 (***)
	Residual	3150	4	787.5		

12. Supplementary Figures

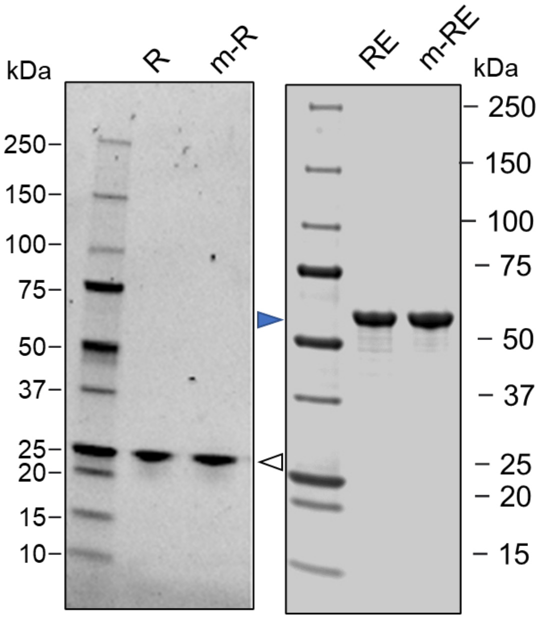

Figure S1. Characterization of purified recoverin-containing constructs using SDS-PAGE.

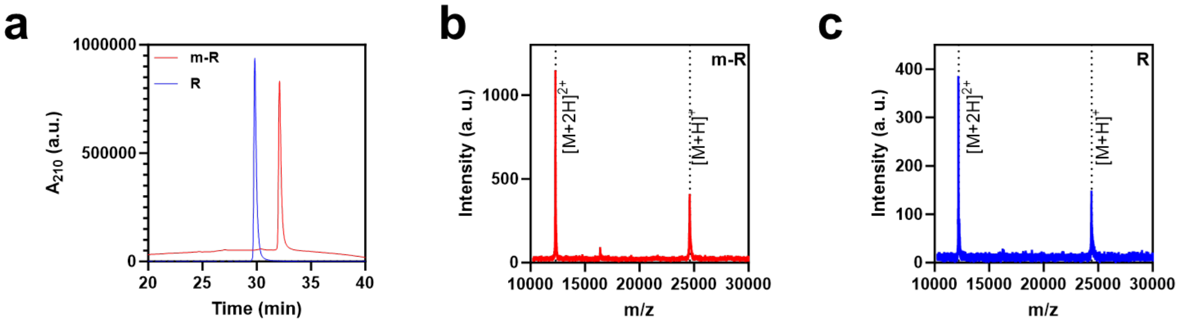

Figure S2. Characterization of recoverin controls (Rc) using liquid chromatography and mass spectrometry. a) RP-HPLC confirms increased retention time for myristoylated R, consistent with the increased hydrophobicity. **b,c)** MALDI-TOF analysis shows a 210 Da increase in molecular weight upon myristoylation. Vertical lines are added to denote the expected m/z ratio for single- and double-charged ions.

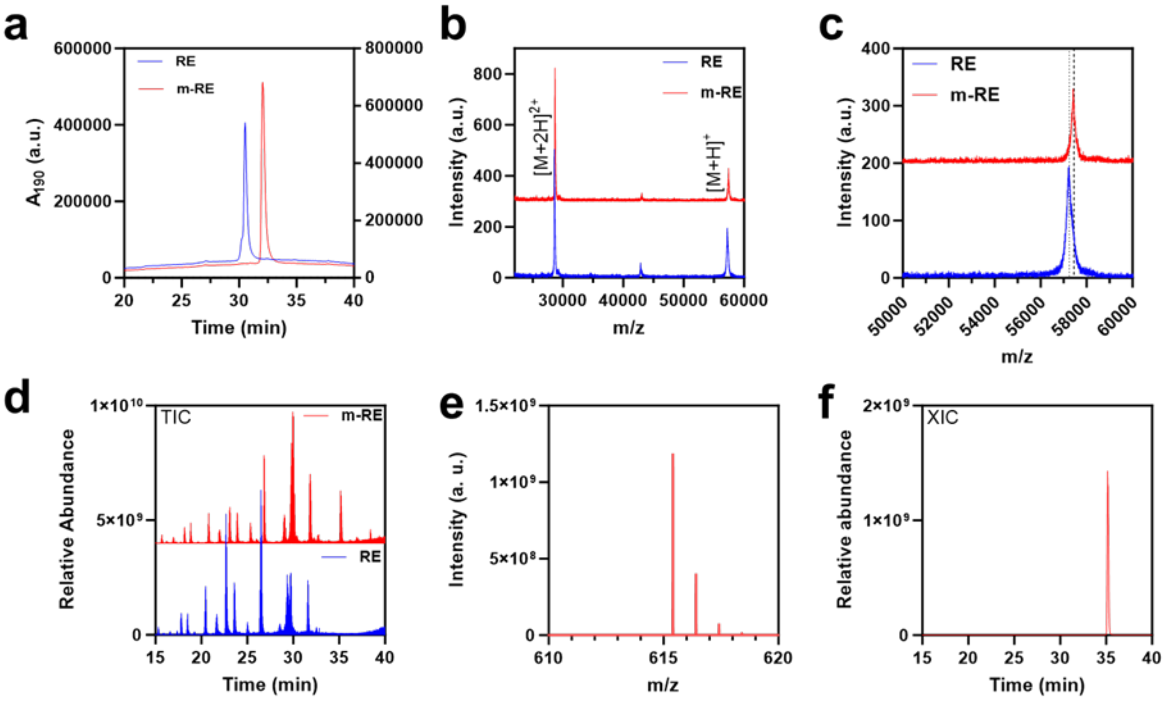

Figure S3. Liquid chromatography and mass spectrometry of recoverin fusions. a) RP-HPLC analysis of unmodified and myristoylated RE fusion proteins. The retention time of myristoylated constructs is increased, indicating higher hydrophobicity of the lipidated isoform. **b, c)** MALDI-TOF analysis of RE fusions. The vertical dotted and dashed lines in (c) represent the theoretical molecular weight of unmodified and lipidated isoforms, respectively. **d)** LC-MS analysis of trypsin digests reveals a distinct peak for a hydrophobic peptide fragment (~35 min) present only in myristoylated RE. **e)** The isotope pattern of the peak at 35 min matches the myristoylated N-terminal peptide of RE, myr-GNSK. **f)** The extracted-ion chromatogram highlights the specific signal from trypsinized myristoylated RE.

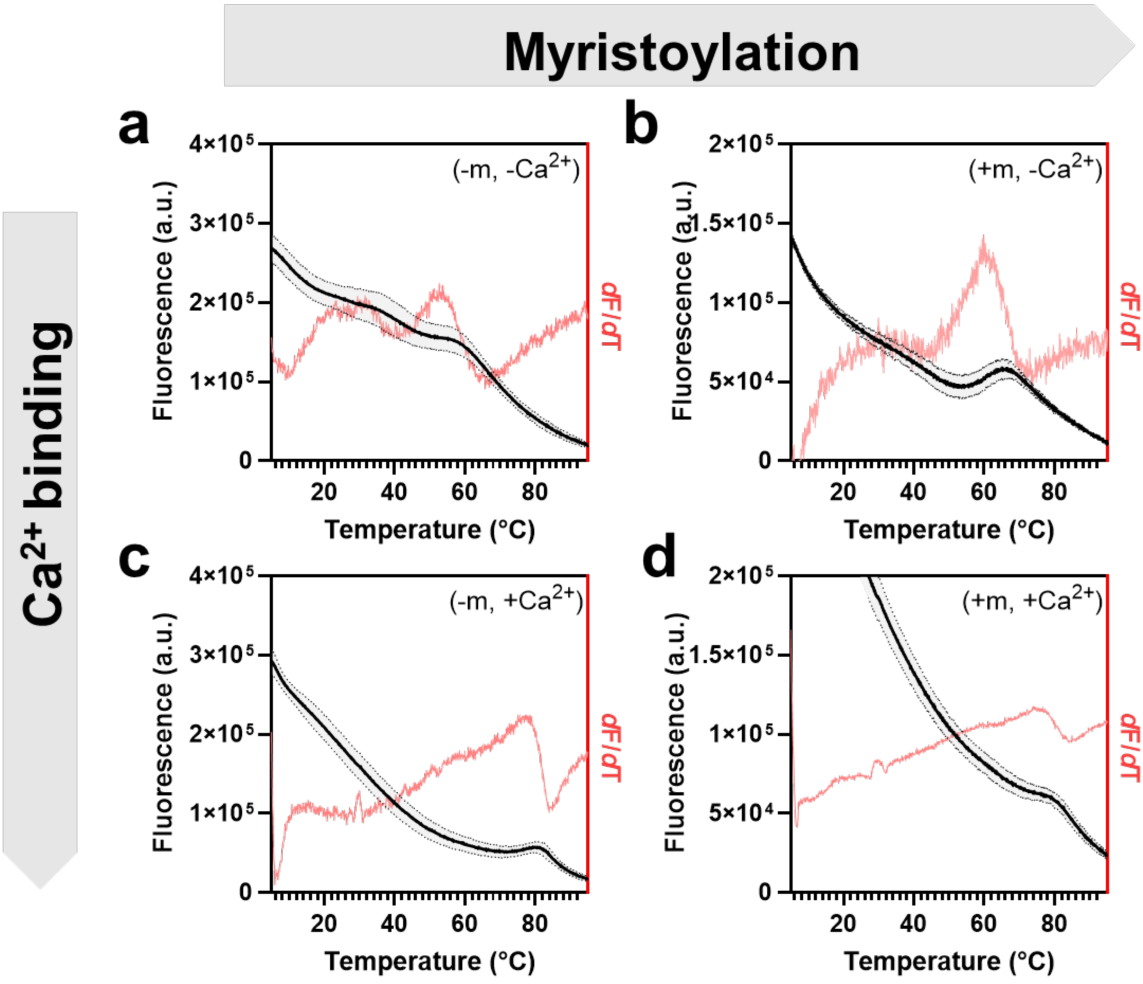

Figure S4. Thermal shift assay of Recoverin. a) Without myristoyl or calcium; **b)** with myristoyl alone; **c)** with calcium alone; and **d)** with both myristoyl and calcium. The solid black line and shaded area represent the mean and standard deviation of three measurements. In the absence of myristoyl and calcium (a), the temperature-dependent fluorescence peak is noticeably broader compared to other conditions, and exhibits notable shoulder around 30°C. This could be due to the association of SYPRO Orange with the hydrophobic residues of the myristoyl binding site[6] or due to intermolecular association of recoverin proteins, which has been previously observed for R(-m, -Ca^{2+}).[7] The transitions observed in other cases closely match the melting temperatures for recoverin determined using various biophysical assays as reported in the literature.[8,9]

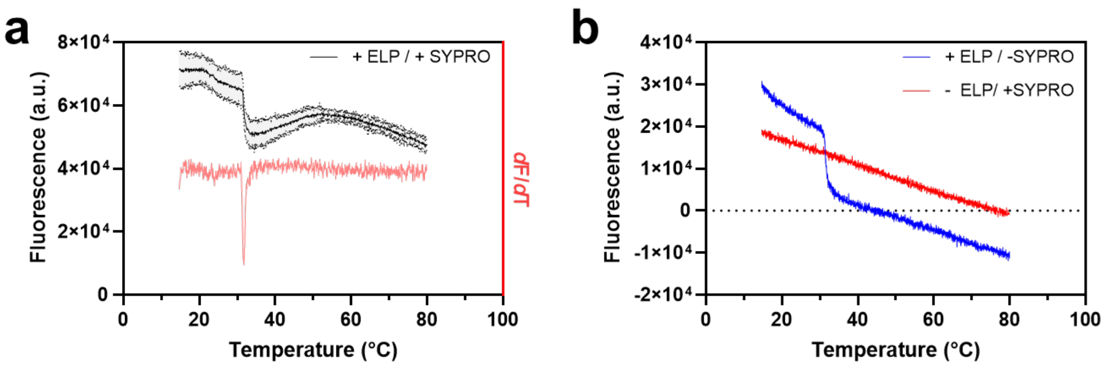

Figure S5. Thermal shift assay of ELP and control experiments. **a)** Fluorescence of SYPRO Orange mixed with ELP slightly decreases above the transition temperature of Ec, indicating minimal change in the microenvironment of SYPRO upon phase-separation of ELP. The dashed line and the shaded areas represent mean and standard deviation of three measurements. **b)** Control experiments using ELP alone (blue line) or SYPRO Orange alone (red line) indicate that the slight decrease in signal intensity in (a) is likely due to the increased turbidity of the solution due to the light scattering from large coacervates formed by ELP. This suggests that the interaction between SYPRO and ELP does not change significantly as a function of temperature. Therefore, SYPRO Orange was used as a probe to monitor the transition of the R-domain as a function of temperature.

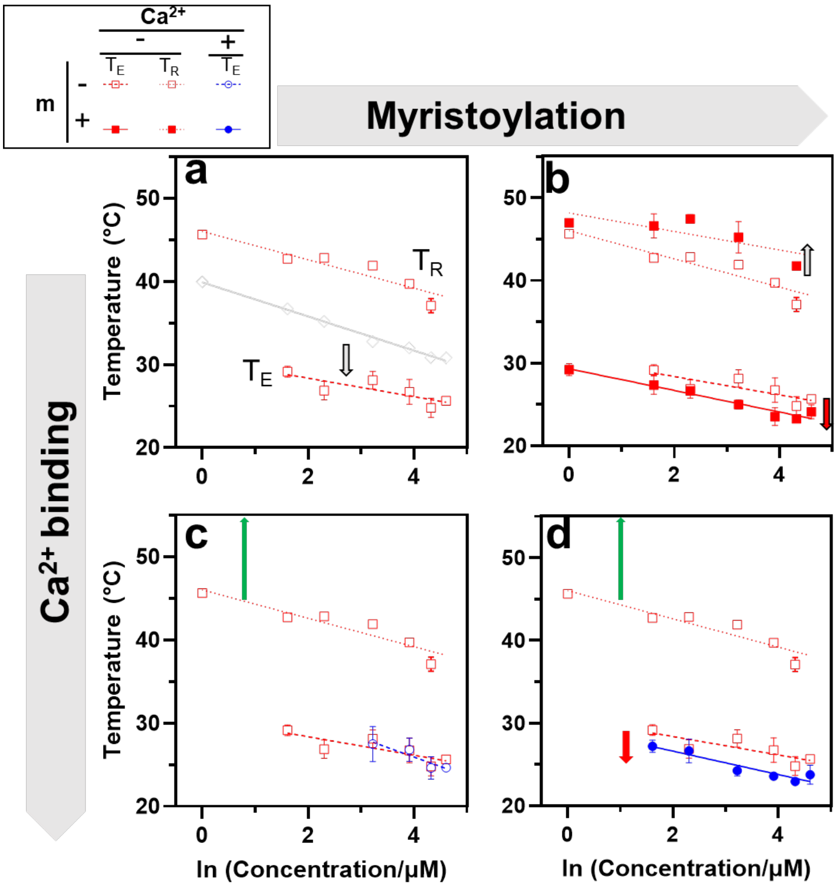

Figure S6. Differential effects of myristoylation and calcium on concentration dependencies of RE transitions. Four panels illustrate how various factors influence critical temperatures (T_E for E-domain, T_R for R-domain): **a)** Fusion effect [E vs. RE] without calcium or myristoylation (-m, -Ca^{2+}). Gray line corresponds to transition temperature for E_c (i.e., T_t for ELP alone); **b)** Impact of myristoylation (± m) without calcium (-Ca^{2+}); **c)** Influence of calcium (± Ca^{2+}) without myristoylation (-m); **d)** Combined effects of myristoylation and calcium (-m, -Ca^{2+}) vs. (+m, +Ca^{2+}).

Supplementary note. The phase behavior of chimeric polypeptides is altered by recoverin's lipidation or calcium binding. We present these results across multiple panels for a thorough evaluation:

- Initially, we assessed ELP fusion's impact by comparing RE to E in the absence of calcium (Figure S6a). These results suggest that fusion of R and E alters the properties of both protein domains: 1) by lowering the T_E of E-domain and reducing its concentration dependence compared to Ec (gray arrow); 2) rendering the R-domain more susceptible to unfolding and aggregation at higher temperatures. We attribute the reduction in T_E to the presence of a hydrophobic patch in recoverin near the fusion site that alters the hydration of ELP.[10] As the temperature is increased above the T_E, the dehydration and aggregation of the E-domain may facilitate inter-molecular aggregations of recoverin at elevated temperatures (i.e., reducing the T_R).
- Figure S6b compares the behavior of RE with m-RE to highlight the impact of myristoylation in the absence of calcium. First, even though the myristoyl group is attached to Recoverin, and not to the ELP, even when it is sequestered inside recoverin, it still alters the hydration and phase separation of the appended ELP, presumably by altering the hydration and surface properties of recoverin (red arrow).[7] Second, in the absence of calcium, myristoylation increases R-domains' T_R and alters its concentration dependence (gray arrow). This indicates that m-RE is more thermally stable than RE, and its unfolding likely occurs through a different mechanism, such as a cooperative intramolecular event.
- Unlike lipidation that affected both domains, binding of calcium to RE increases the thermal stability of the R-domain only as the second transition disappears (Figure S5c, green arrow), while having minimal effect on the E-domain. When myristoylation and calcium binding are combined (Figure S6d), their effect on phase separation is combined as well. Myristoylation reduces the T_E of the ELP-domain (red arrow), and both factors increase T_R (i.e., stabilize recoverin and increase its thermal stability, green arrow). However, calcium binding appears to play a more significant role in stabilizing the structures compared to myristoylation. Together, these experiments demonstrate that the liposwitching capability of recoverin modulates the temperature-dependent phase behavior of the fusion protein.

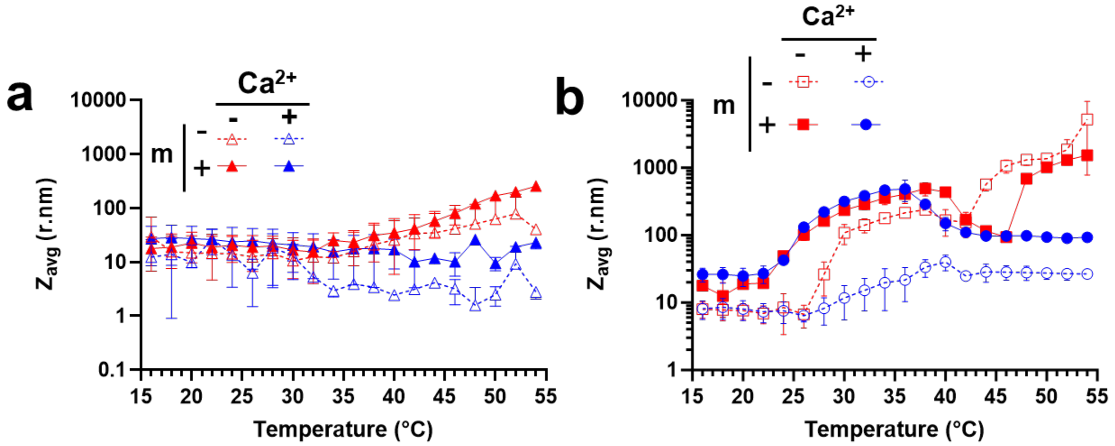

Figure S7. Untransformed variable-temperature DLS data for R and RE fusions in presence or absence of myristoylation (± m) and calcium (± Ca^{2+}). Temperature-dependent assembly of RE fusions is influenced by liposwitching. **a)** The untransformed DLS data shows that the size of R-domain assemblies is similar, regardless of myristoylation or calcium binding. **b)** The transformed DLS data further confirms that the myristoyl-switching of the R-domain does not significantly influence its self-assembly. These results emphasize that the distinct emergent assembly observed in RE fusions (Figure 4b) is primarily driven by the interaction between the R-domain and E-domain.

a

b

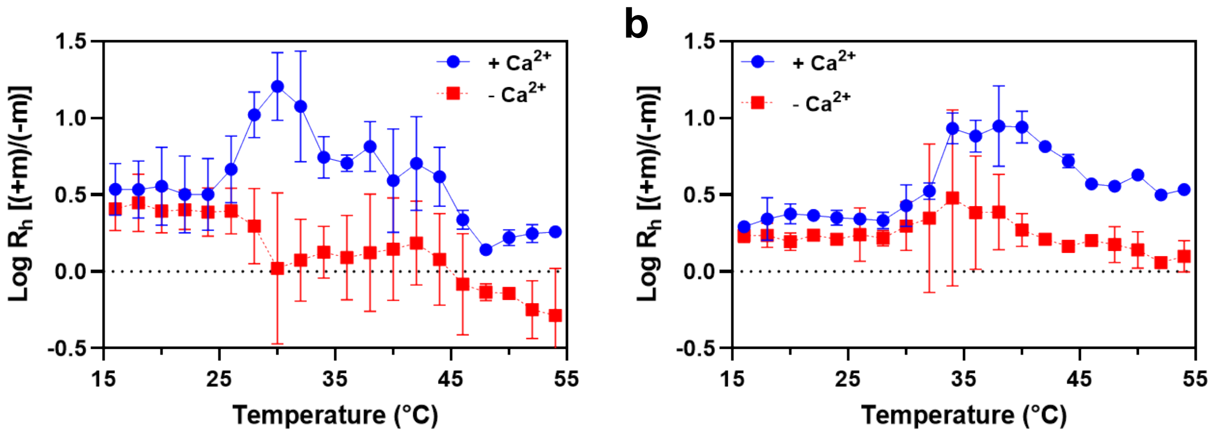

Figure S8. Transformed DLS results showing liposwitching temperature threshold can be altered by changing the composition of the E-domain. a) RE' and **b)** RE''. Protein sequences are reported on page S6.

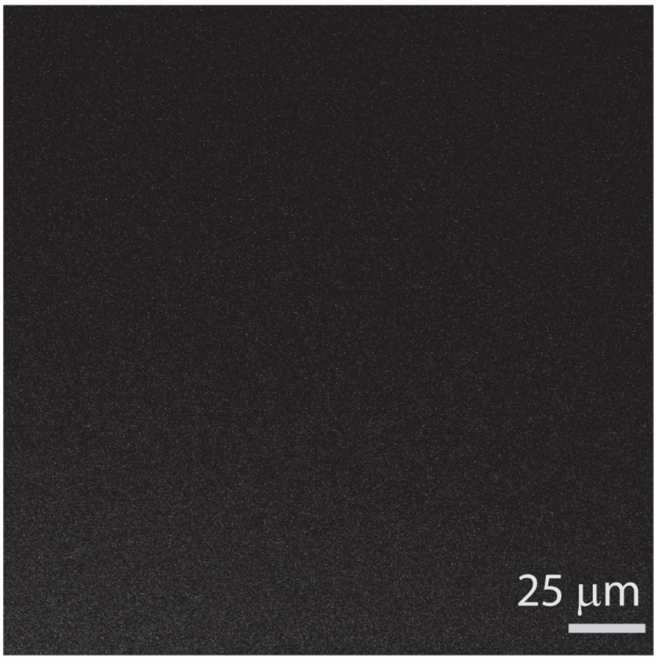

Figure S9. Visualization of RE(-m, +Ca^{2+}) using confocal microscopy at 50°C. RE assemblies under these conditions remain below the resolution limit of the microscope.

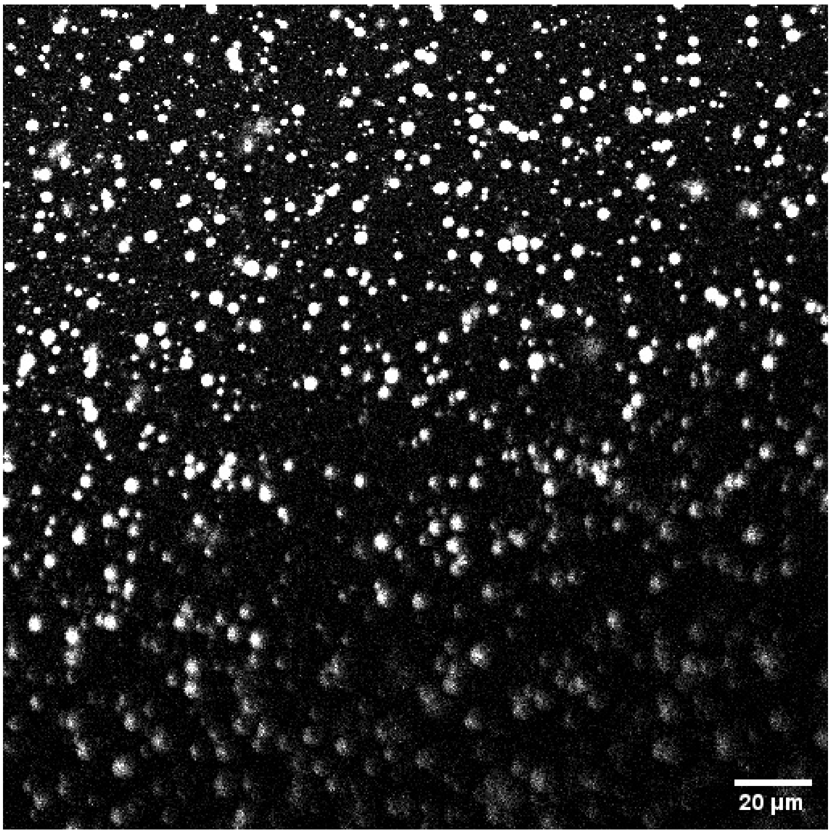

Figure S10. Visualization of ELP control (Ec) coacervates using confocal microscopy. ELP forms canonical liquid-like coacervates with spherical morphology above its transition temperature.

13. References

1. McDaniel, J. R.; Mackay, J. A.; Quiroz, F. G.; Chilkoti, A., Recursive directional ligation by plasmid reconstruction allows rapid and seamless cloning of oligomeric genes. *Biomacromolecules* **2010,** *11* (4), 944-952.
2. Hossain, M. S.; Ji, J.; Lynch, C. J.; Guzman, M.; Nangia, S.; Mozhdehi, D., Adaptive Recombinant Nanoworms from Genetically Encodable Star Amphiphiles. *Biomacromolecules* **2022,** *23* (3), 863-876.
3. Desmeules, P.; Penney, S. E.; Salesse, C., Single-step purification of myristoylated and nonmyristoylated recoverin and substrate dependence of myristoylation level. *Anal. Biochem.* **2006,** *349* (1), 25-32.
4. Neubert, T. A.; Walsh, K. A.; Hurley, J. B.; Johnson, R. S., Monitoring calcium-induced conformational changes in recoverin by electrospray mass spectrometry. *Protein Sci.* **1997,** *6* (4), 843-50.
5. Meyer, D. E.; Chilkoti, A., Purification of recombinant proteins by fusion with thermally-responsive polypeptides. *Nat. Biotechnol.* **1999,** *17* (11), 1112-5.
6. Marino, V.; Riva, M.; Zamboni, D.; Koch, K.-W.; Dell'Orco, D., Bringing the Ca2+ sensitivity of myristoylated recoverin into the physiological range. *Open Biology* **2021,** *11* (1), 200346.
7. Kataoka, M.; Mihara, K. i.; Tokunaga, F., Recoverin Alters Its Surface Properties Depending on Both Calcium-Binding and N-Terminal Myristoylation1. *J. Biochem.* **1993,** *114* (4), 535-540.
8. Potvin-Fournier, K.; Lefèvre, T.; Picard-Lafond, A.; Valois-Paillard, G.; Cantin, L.; Salesse, C.; Auger, M., The Thermal Stability of Recoverin Depends on Calcium Binding and Its Myristoyl Moiety As Revealed by Infrared Spectroscopy. *Biochemistry* **2014,** *53* (1), 48-56.
9. Permyakov, S. E.; Cherskaya, A. M.; Senin, II; Zargarov, A. A.; Shulga-Morskoy, S. V.; Alekseev, A. M.; Zinchenko, D. V.; Lipkin, V. M.; Philippov, P. P.; Uversky, V. N.; Permyakov, E. A., Effects of mutations in the calcium-binding sites of recoverin on its calcium affinity: evidence for successive filling of the calcium binding sites. *Protein Eng.* **2000,** *13* (11), 783-90.
10. Trabbic‐Carlson, K.; Meyer, D.; Liu, L. a.; Piervincenzi, R.; Nath, N.; LaBean, T.; Chilkoti, A., Effect of protein fusion on the transition temperature of an environmentally responsive elastin‐like polypeptide: a role for surface hydrophobicity? *Protein Eng. Des. Sel.* **2004,** *17* (1), 57-66.
